# A Critical Evaluation of the Down Syndrome Diagnosis for LB1, Type Specimen of *Homo floresiensis*

**DOI:** 10.1371/journal.pone.0155731

**Published:** 2016-06-08

**Authors:** Karen L. Baab, Peter Brown, Dean Falk, Joan T. Richtsmeier, Charles F. Hildebolt, Kirk Smith, William Jungers

**Affiliations:** 1 Department of Anatomy, Arizona College of Osteopathic Medicine, Midwestern University, 19555 N. 59th Avenue, Glendale, AZ, 85308, United States of America; 2 Bioanthropology, School of Archaeology & Anthropology, Australian National University, Acton, ACT, 2601, Australia; 3 Department of Anthropology, Florida State University, Tallahassee, FL, 32306–7772, United States of America; 4 School for Advanced Research, Santa Fe, NM, 87505, United States of America; 5 Department of Anthropology, Pennsylvania State University, University Park, PA, 16802, United States of America; 6 Department of Radiology, Mallinckrodt Institute of Radiology, Washington University School of Medicine, 510 South Kingshighway, St. Louis, Missouri, 63110, United States of America; 7 Department of Biomedical Informatics, University of Arkansas for Medical Sciences, Little Rock, AR, 72205, United States of America; 8 Department of Anatomical Sciences, School of Medicine, Stony Brook University, Stony Brook, NY, 11794–8081, United States of America; 9 Association Vahatra, BP 3972, Antananarivo 101, Madagascar; University of Wisconsin, UNITED STATES

## Abstract

The Liang Bua hominins from Flores, Indonesia, have been the subject of intense scrutiny and debate since their initial description and classification in 2004. These remains have been assigned to a new species, *Homo floresiensis*, with the partial skeleton LB1 as the type specimen. The Liang Bua hominins are notable for their short stature, small endocranial volume, and many features that appear phylogenetically primitive relative to modern humans, despite their late Pleistocene age. Recently, some workers suggested that the remains represent members of a small-bodied island population of modern Austro-Melanesian humans, with LB1 exhibiting clinical signs of Down syndrome. Many classic Down syndrome signs are soft tissue features that could not be assessed in skeletal remains. Moreover, a definitive diagnosis of Down syndrome can only be made by genetic analysis as the phenotypes associated with Down syndrome are variable. Most features that contribute to the Down syndrome phenotype are not restricted to Down syndrome but are seen in other chromosomal disorders and in the general population. Nevertheless, we re-evaluated the presence of those phenotypic features used to support this classification by comparing LB1 to samples of modern humans diagnosed with Down syndrome and euploid modern humans using comparative morphometric analyses. We present new data regarding neurocranial, brain, and symphyseal shape in Down syndrome, additional estimates of stature for LB1, and analyses of inter- and intralimb proportions. The presence of cranial sinuses is addressed using CT images of LB1. We found minimal congruence between the LB1 phenotype and clinical descriptions of Down syndrome. We present important differences between the phenotypes of LB1 and individuals with Down syndrome, and quantitative data that characterize LB1 as an outlier compared with Down syndrome and non-Down syndrome groups. *Homo floresiensis* remains a phenotypically unique, valid species with its roots in Plio-Pleistocene *Homo* taxa.

## Introduction

Interpretation of the Late Pleistocene Flores hominins has been controversial since their initial description in 2004 as a distinct species, *Homo floresiensis* [1]. The many phylogenetically primitive features observed in the Flores specimens relative to modern humans suggest that their ancestry is rooted in Plio-Pleistocene *Homo*, perhaps *H*. *erectus* or *H*. *habilis*. Since its discovery, workers have confirmed that cranial and endocranial shape and many cranial characteristics of LB1 correspond to classic *H*. *erectus* characters [2–5], while some postcranial and mandibular features, such as the interlimb proportions and symphyseal shape are more primitive than *H*. *erectus*, and more closely resemble early *Homo* or even australopiths [6–8]. Cladistic analyses placed the origin of *H*. *floresiensis* earlier than the origin of *H*. *erectus* [9, 10], but both a *H*. *erectus* or an early *Homo* origin would imply evolutionary convergences in morphology [11, 12].

At the same time, a minority of workers maintains that the Flores hominins are small-bodied modern humans, and, at least in the case of the most complete specimen, LB1, pathologically altered. Several clinical signs (e.g., microcephaly) and specific pathologies (endemic hypothyroidism, Laron syndrome) have been proposed [13–18] and subsequently rejected [19–24]. Most recently, Henneberg, Eckhardt [25] reported that LB1 manifested many clinical signs of Down syndrome (DS). We provide an overview of the DS phenotype, and re-evaluate the evidence presented by Henneberg, Eckhardt [25] in support of this diagnosis for LB1.

The stratigraphic layers containing *H*. *floresiensis* were initially dated to 95–74 to 12 kyr [1, 26]. This chronology was recently revised to 100 to 60 kyr based on a better understanding of the cave geology and more extensive dating of the *H*. *floresiensis* fossils themselves, as well as sediment and speleothems of these layers and overlying tephra [27]. The dates are internally consistent. Dates associated with tools thought to be manufactured by this species may extend the range from 190 to 50 kyr [27, 28]. Modern human remains are not documented in the broader region of Southeast Asia and Australia until 50–45 kyr [29–31]. Thus, to the extent that this new chronology accurately reflects the true dates of occupation of the region, there was no temporal overlap between *H*. *floresiensis* and *H*. *sapiens* on Flores.

**Fig 1:**
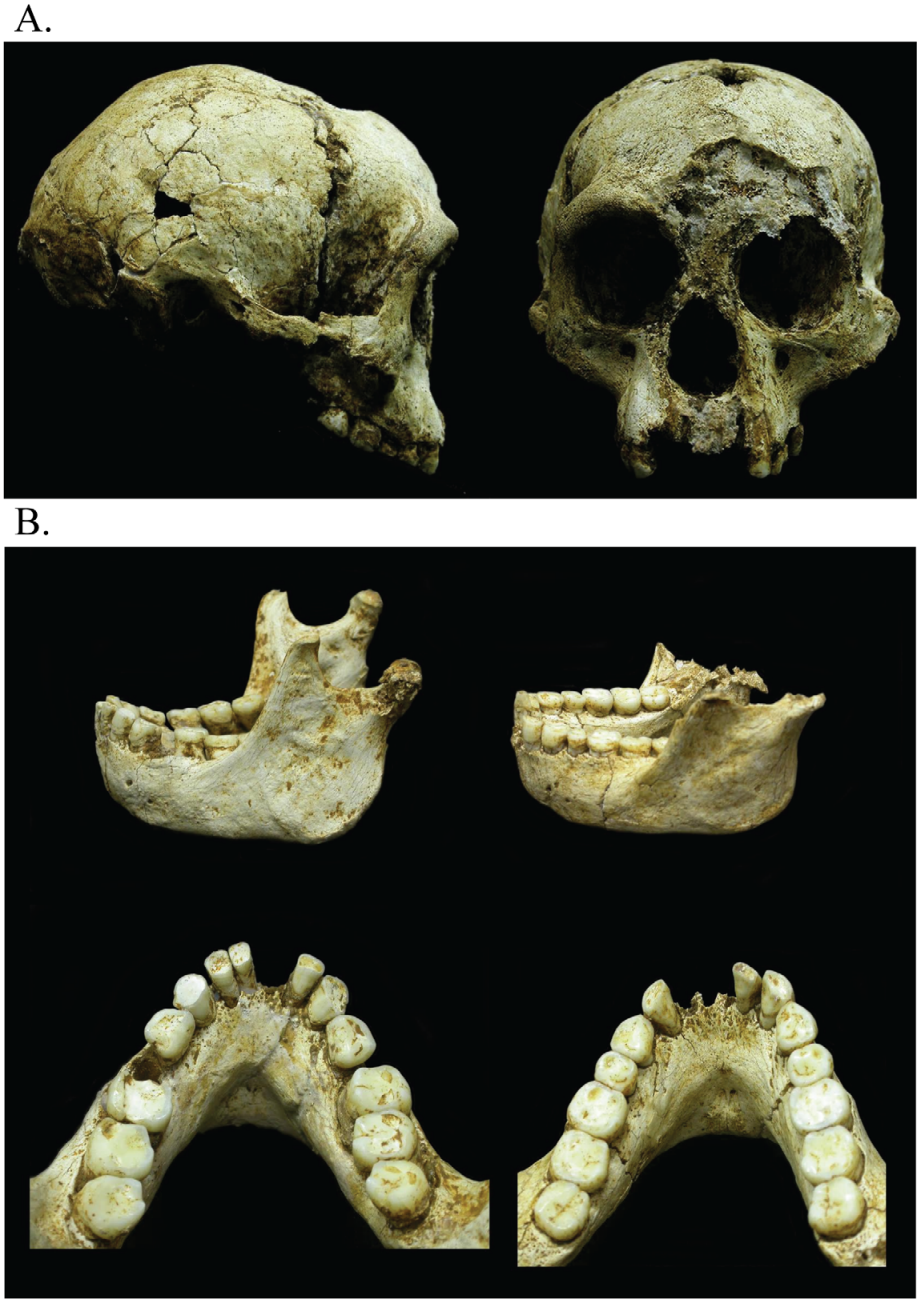
Photographs of LB1 cranium and LB1 and LB6 mandibles. (A) The cranium is shown in right lateral and anterior views. (B) The LB1 (left) and LB6 (right) mandibles are shown in left lateral and occlusal views.

The Liang Bua remains consist, in part, of a fairly complete skeleton with a cranium and mandible, assigned to LB1. LB1 is the holotype for the species *Homo floresiensis* and presents the only cranium thus far recovered [1] (Fig 1). The LB6 individual presents additional evidence in the form of a mandible and numerous postcranial bones [1, 26, 32, 33]. The mandibular anatomy of LB1 and LB6 are quite comparable [8] (Fig 1), and the postcranial elements of BL6 and other individuals from the site further confirm that this population was small-bodied.

### Down syndrome and paleopathology

Down syndrome, described in 1866 by John Langdon Down [34], is caused by trisomy of some or all of chromosome 21 (and is also known as Trisomy 21) through non-disjunction or, more rarely, translocation, and is seen in 1/600-1/2000 live births [35]. More than 100 clinical signs are reported to be associated with DS, many of which are used in fetal diagnosis. Although the combination of many clinical features are characteristic of DS, a valid diagnosis requires karyotype, an option that is not available in the case of the subfossils from Flores. Many of the clinical signs of DS are soft tissue characteristics which cannot be assessed in fossils, such as a single palmar crease, up-slanting palpebral fissures, and a protruding tongue. DS is a highly heterogeneous condition and although skeletal features are noted (brachycephaly, maxillary retrusion), there is no single, diagnostic skeletal phenotype present in all individuals with DS that distinguishes these individuals from the rest of the population. Finally, many of the clinical signs of DS are not unique to this syndrome but are present in other syndromes (e.g., midfacial hypoplasia, microcephaly) or, in some cases, are part of the normal range of variation in euploid (having a balanced set of chromosomes) individuals (e.g., flat foot, occlusal abnormalities, Brushfield spots, cardiac septation defects) (see also [36]). Chen [37] advises that ≥8 characteristic clinical findings of DS should be present for a clinical DS diagnosis, but that chromosomal analysis is preferred and necessary in uncertain cases. Therefore, a large number of DS signs must be positively identified if a definite diagnosis of DS is to be accepted. Major reference works on paleopathology in osteological remains provide limited assistance as they either do not address DS explicitly [38, 39] or do not indicate which skeletal features in isolation or combination are sufficient for a differential diagnosis [40].

This type of analysis is not, however, without precedent. A 7200-year-old skeleton from the site of Santa Rosa Island, CA was notable in its metopism, flat cranial base, wide interorbital distance, reduced facial height, dental anomalies and small postcranial elements (estimated stature of 154 cm). The authors noted that these features were consistent with four syndromes including DS, and concluded that “This case illustrates…some of the difficulties of diagnosing craniofacial syndromes in prehistoric skeletal remains” ([41], p. 179). Rivollat et al. [42] identified a child from the French medieval site of Saint-Jean-des-Vignes as having DS on the basis of craniofacial and dental characteristics. Rivollat et al. [42] identified differences in the Saint-Jean-des-Vignes skull relative to a non-pathological reference sample and then assessed the differences with descriptions and measurements of DS available from the clinical literature. A single individual from Tauberbischofsheim, Germany was diagnosed with DS by Czarnetzki et al. [43] out of the >7000 examined in the time period 3200 BC-AD 800 in Europe. They used five 2D landmarks from the median facial profile, but did not provide images or methodological details, including evidence that this facial profile was distinct from other disorders with midfacial hypoplasia (i.e., Achondroplasia or Crouzon syndrome). The most thorough archaeological diagnosis of DS was published by Brothwell [44], who identified a probable case of DS in a Saxon child based on comparisons of craniodental traits with other member of its population and comparative DS material. Such traits as microcephaly, hyperbrachycephaly, thin cranial bones, small maxilla but not mandible and dental irregularities were most concordant with DS. Although the author considers the upright basi-occipital to be indicative of DS, this feature, along with a low spheno-ethmoidal angle, may suggest a highly flexed cranial base, which is actually at odds with the typical DS phenotype. Brothwell ([44], p. 49) emphasizes that “It is important in this type of analysis to consider all the features together, rather than attempt to isolate one special characteristic feature.”

The present study differs from these studies in that it does not seek to make a clinical diagnosis but rather to refute an existing diagnosis. Nevertheless, a similar comparative approach, evaluating morphological features of LB1 against both the DS and euploid phenotypes with an emphasis on those features that most clearly differentiate these two groups and are found at a high frequency in DS, is used here. Many, but not all, of these traits were also discussed by Henneberg, Eckhardt [25]. Yet, this is an imperfect approach as we have previously described and quantified the many ways in which LB1 does not fit within the modern human range of variation but is rather aligned more closely with archaic *Homo*. One problem this poses is that LB1 may not fit within either the modern euploid or DS range of variation and instead be an outlier. Conversely, it is possible that LB1 could converge on features in DS for reasons that are not related to a chromosomal abnormality. For example, LB1 converges in some aspects of cranial shape with humans exhibiting extreme microcephaly due to its probably ancestry from a small-brained early *Homo* species [21]. These issues must be born in mind while interpreting the results of the comparative analyses presented below.

### Down syndrome and LB1

The presence of some characteristic features of DS in LB1 is insufficient to confirm a diagnosis of DS, and, conversely, the absence of some signs is insufficient to reject a DS diagnosis. Rather, we must be satisfied to judge the likelihood of DS based on assessment of those DS signs that are evaluable in the hard tissues preserved for LB1. The absence of all or most of these signs would indicate a lowered probability of DS. A summary of features seen more commonly in individuals with DS than in the general population that are potentially discernable in skeletal or dental remains are summarized in Table 1. Only those dental features specifically mentioned by Henneberg et al [25] are included in Table 1, but additional dental traits are listed in S1 Table. In many cases these signs were identified and evaluated at the fetal, newborn or juvenile stage, and it is not clear that they remain valid markers of the adult DS phenotype. For example, brachymesophalangia-5 is more common in young individuals with DS and there is some evidence that this condition may become normalized during subsequent growth and development [45, 46]. Similarly, the flaring ilia of the pelvis are also more diagnostic at fetal and infant stages than in adults with DS [47, 48].

**Table 1:** Features Associated with Down syndrome that may be evaluable in hard tissues and/or were discussed by Henneberg et al. (2014).

Features	Our Notes	Status in LB1 as per Henneberg et al. (2014)	Status in LB1 as per current study
**Brain**			
Brain smaller than matched population / sex averages		Yes	LB1 is outside of the DS range
Small cerebellum		Yes	All endocast dimensions are small
**Growth and Development**			
Height and weight 2–4 SD below general population postnatally		Yes (short stature)	LB1 is outside of the DS range
Delayed osseous maturation			
**Craniodental**			
Increased levels of fluctuating asymmetry of face / dentition	Must be measured at the *population* level	Yes (directional asymmetry of facial skeleton)	Cannot be assessed at individual level (S1 Text)
Microcephaly		Yes	
Persistent metopic suture	67% of males; 42% of females [35]		
Flat occiput	76% of children and adults [35]		
Brachycephaly	75% of Danish males 19–25 years [49]	Yes	LB1 is outside of the DS range
Absent frontal sinus	83–86% of children and adults [50, 51]	Yes	Probable
Poorly pneumatized sphenoid sinus	~66% of children and adults [51, 52]	Yes	Sinus present, size uncertain
Hypoplastic maxillary sinus	7% of Saudi mixed-sex sample 12–24 years [50]; 45% of children 4–15 years [53]		No
Midfacial hypoplasia		Yes (underdeveloped maxilla)	No
Flat nasal bridge	61% of children and adults [35]		Cannot be evaluated
Narrow palate	67% of children and adults [35]		No
Occlusal problems (e.g., mandibular overjet, anterior open bite)			No
Irregular alignment of teeth	71% of children and adults [35]	Yes	
Periodontal disease	>90% [35]	Yes	Yes
Missing teeth (including M3 / excluding M3)	92% / 56% [54]	Yes	Yes
Taurodontism	55–86% [55–57]	Yes	No (where it can be evaluated; S1 Text and S1 Table)
Thin cranial bones	59% of Saudi mixed-sex sample 12–24 years [50]		No
Microgenia		Yes	Cannot be evaluated (S1 Text)
Flat cranial base angle (platybasia)	140.3 in DS; 129.9 in controls (<18 years); 144.0° in DS; 135.6° in controls (15–18 years) [58, 59]		No (<130°)
Plagiocephaly	Not usually listed as an adult DS feature	Yes	Yes, but irrelevant to DS diagnosis
**Postcranial**			
Flexible flatfoot due to ligamentous laxity	60% of children 4–10 years [60]; 70% of adults [61]	Yes	Cannot be evaluated (S1 Text)
Atlantoaxial instability due to ligamentous laxity	10–20% [35]	Yes	Cannot be evaluated but unlikely (S1 Text)
Short femur	Common prenatal marker for DS; not usually listed as an adult DS feature	X (relative to foot and arm)	LB1 is outside of the DS range
Dorsolumbar kyphosis	11% of children and adults [35]		
Short broad hands	70% of children and adults [35]		
Brachydactyly	67% of children and adults [35]	Yes	Some distal phalanges short in LB1 & LB6
Clinodactyly	59% of children and adults [35]		
Brachymesophalangia (shortening of middle phalanx of fifth finger)	59% of children and adults [35]		
Arthritis, including juvenile rheumatoid arthritis-like arthropathy	<1% [62, 63]	Yes	No pathology in carpal bones [64, 65]
Metatarsus primus varus	40% of children 4–14 years [60]		No (S1 Text)
Hallux valgus	26% of children 4–14 y [60]		Cannot be evaluated (S1 Text)
Gap between hallux and second toe	50% of children and adults [35]		Cannot be evaluated (S1 Text)
Flaring iliac wings	38% of adults [48]	Yes	
**Other**			
Hypothyroidism	1.5–6.1% of children [66]; 19 cases of hypothyroidism in a 15 year longitudinal study of 112 adults with DS [67]; 0.7–23.5% of newborns had congenital hypothyroidism [68, 69]	Yes	No [19, 21]

The identification of LB1 as exhibiting a DS phenotype was previously rebutted by Westaway, Durband [70] with regard to the mandibular evidence. Additional preliminary critiques were offered by Argue [71] and Baab et al. [72]. Here we provide comparative analyses of linear and volumetric measurements of the endocast, neurocranial and symphyseal shape, stature, intralimb proportions and digit length among samples of individuals with a documented diagnosis of DS, samples of euploid individuals, and LB1. We also provide new images derived from CT data that shed light on the presence of cranial sinuses and re-evaluate the evidence regarding the foot:femur (foot:thigh) ratio in LB1 and individuals with DS. Some additional features discussed by Henneberg, Eckhardt [25] were re-evaluated using the extensive DS literature and results are presented in the accompanying Supplemental Information. This re-evaluation reveals the improbability of a diagnosis of DS based on a synthesis of the clinical literature on DS with comparative analyses of new morphometric evidence from LB1.

## Results

### Brain, Skull and Dentition

#### Brain size

**Fig 2:**
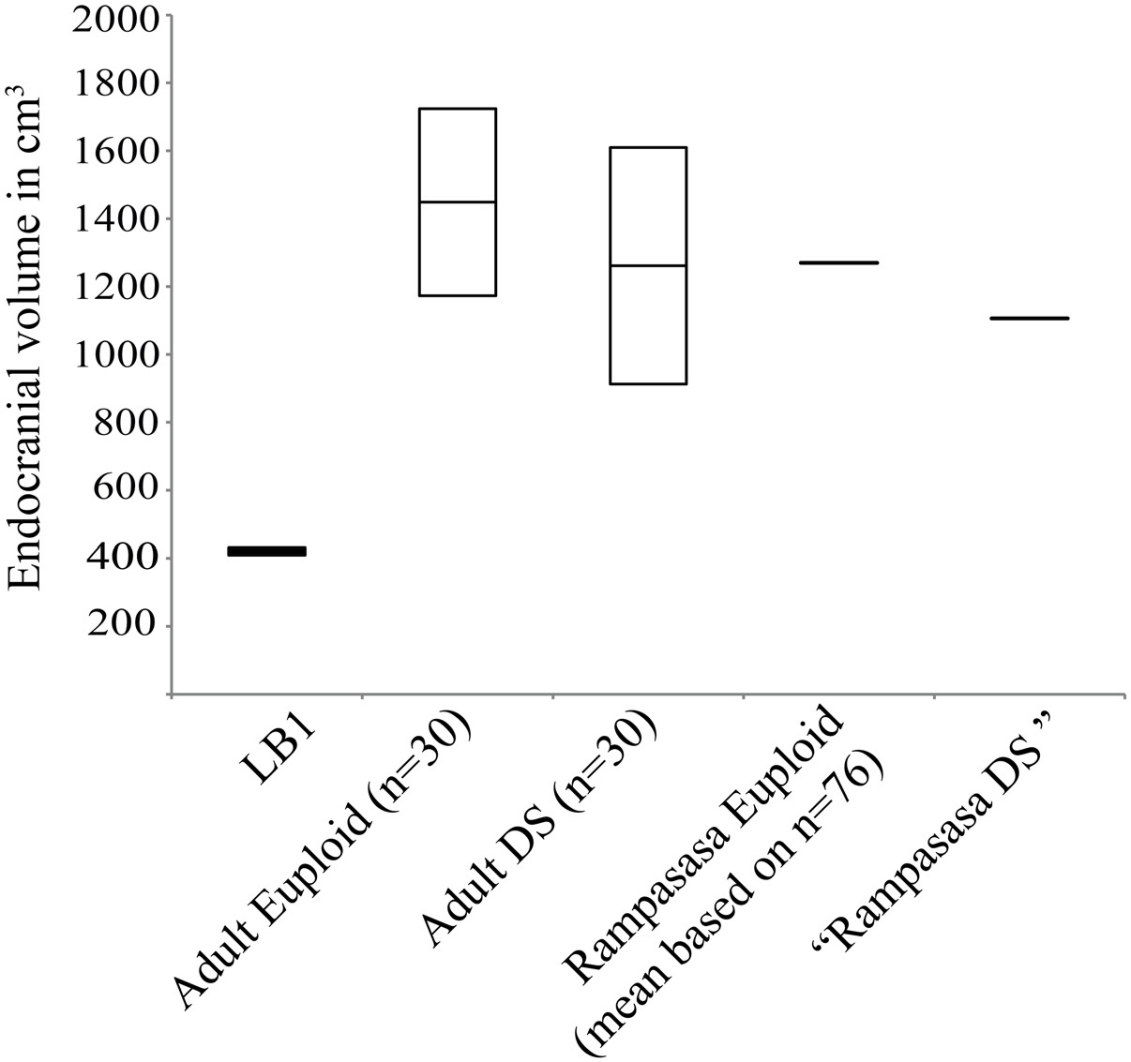
Box plot of endocranial volumes. The DS and matched euploid clinical samples are from Aylward, Habbak [73]. The center line represents the average value while the box captures ± 2 SDs. The mean value for the Rampasasa population is from Henneberg, Eckhardt [25]. The average value of an individual with DS from the Rampasasa population (“Rampasasa DS”) was estimated as 87% of the euploid value, based on the relationship between the matched DS and euploid samples in Aylward, Habbak [73].

Henneberg et al. ([25], p. 1 of SI) stated that “the brain of LB1 is very small, its vault is low, its shape is brachycephalic, and its cerebellar region is small. All of these attributes…are common attributes of individuals with Down syndrome…” The endocranial volume (EV) and linear dimensions of the virtual endocast of LB1 were significantly smaller than our sample of 6 female DS patients (Table 2) and the EV was also significantly smaller than a larger mixed-sex sample of 30 adults (19 males, 11 females) reported by Aylward, Habbak [73] (Fig 2). The mean cranial volume for our six DS subjects was 1174.17 cm^3^ (SD = 132.35; range: 918–1276), which was not significantly different [*t* (34) = -1.154, *p* = 0.256)] from the mean EV of 30 adult DS subjects of 1261.26 cm^3^ (SD = 174.18 cm^3^) reported by Aylward, Habbak [73], indicating that our smaller sample is representative of DS.

**Table 2:** Descriptive statistics and comparisons of the values for the LB1 endocast with values for our sample of 6 subjects with Down syndrome.

Parameter		Number	Mean	Std Dev	Std Err Mean	Lower 95%	Upper 95%	*P* value*
Endocast length (fp-op, mm)								
	Down syndrome	6	154.75	6.8	2.78	147.61	161.89	0.002
	LB1	1	118.4	.	.	.	.	
Cerebellar width (mm)								
	Down syndrome	6	100.63	3.54	1.44	96.92	104.34	<.001
	LB1	1	76.4	.	.	.	.	
Cerebral width (mm)								
	Down syndrome	6	124.95	5.76	2.3	118.91	131	0.009
	LB1	1	103.4	.	.	.	.	
Frontal breadth								
	Down syndrome	6	102.28	5.06	2.07	96.97	107.6	0.005
	LB1	1	80.1	.	.	.		
Endocranial volume (cm^3^)								
	Down syndrome	6	1174.17	132.35	54.03	1035.28	1313.06	0.002
	LB1	1	417	.	.	.	.	.

Note.―fp-op = frontal pole-occipital pole.

*For each measurement, *P* is the probability of obtaining a result at least as extreme as that observed if the null hypothesis were true that the value for LB1 is not lower than the mean value for our 6 subjects with DS. After statistical levels of significance were corrected by adjusting for the false discovery rate, the adjusted levels to achieve statistical significance ranged from 0.01 for cerebellar width (mm) to 0.05 for cerebral width (mm), with all *P* values in Table 2 being below these levels and remaining statistically significant.

Using the data from Aylward, Habbak [73], the average EV of the DS sample was 87% of the average EV from a matched euploid sample. Applying this relationship to the average EV of 1270 cm^3^ for the local Indonesian population of Rampasasa [17], we estimated an average EV of 1106 cm^3^ for individuals with DS from Rampasasa. This value was >2.5 times the size of the LB1 EV (417–426 cm^3^; [4, 74] (Fig 2). Analyses of head circumference yield comparable results (S1 Text).

Cerebellar hypoplasia is another known clinical sequela of DS [73, 75]. In this case the cerebellar volume is reduced disproportionately relative to other regions of the brain, although some of this is attributable to a loss of cerebellar volume during life [76], which cannot be evaluated on an endocast. Henneberg et al. [25] included “small cerebellum” as a sign of DS present in LB1 based on the work of Vannucci et al. [77]. However, Vannucci et al. [77] evaluated only linear, not volumetric, dimensions. Although LB1 does have a low cerebellar breadth: cerebral breadth ratio, in common with DS individuals, the cerebellum is wide relative to (the cube root of) EV, in common with the euploid but not DS pattern (based on data from Table 2). The smaller cerebellar breadth of LB1 documented here (Table 2) and elsewhere is therefore insufficient to argue for the cerebellar hypoplasia seen in some DS individuals. Moreover, the shape of the LB1 endocast differed from DS and euploid samples (S1 Fig).

#### Neurocranial shape

**Fig 3:**
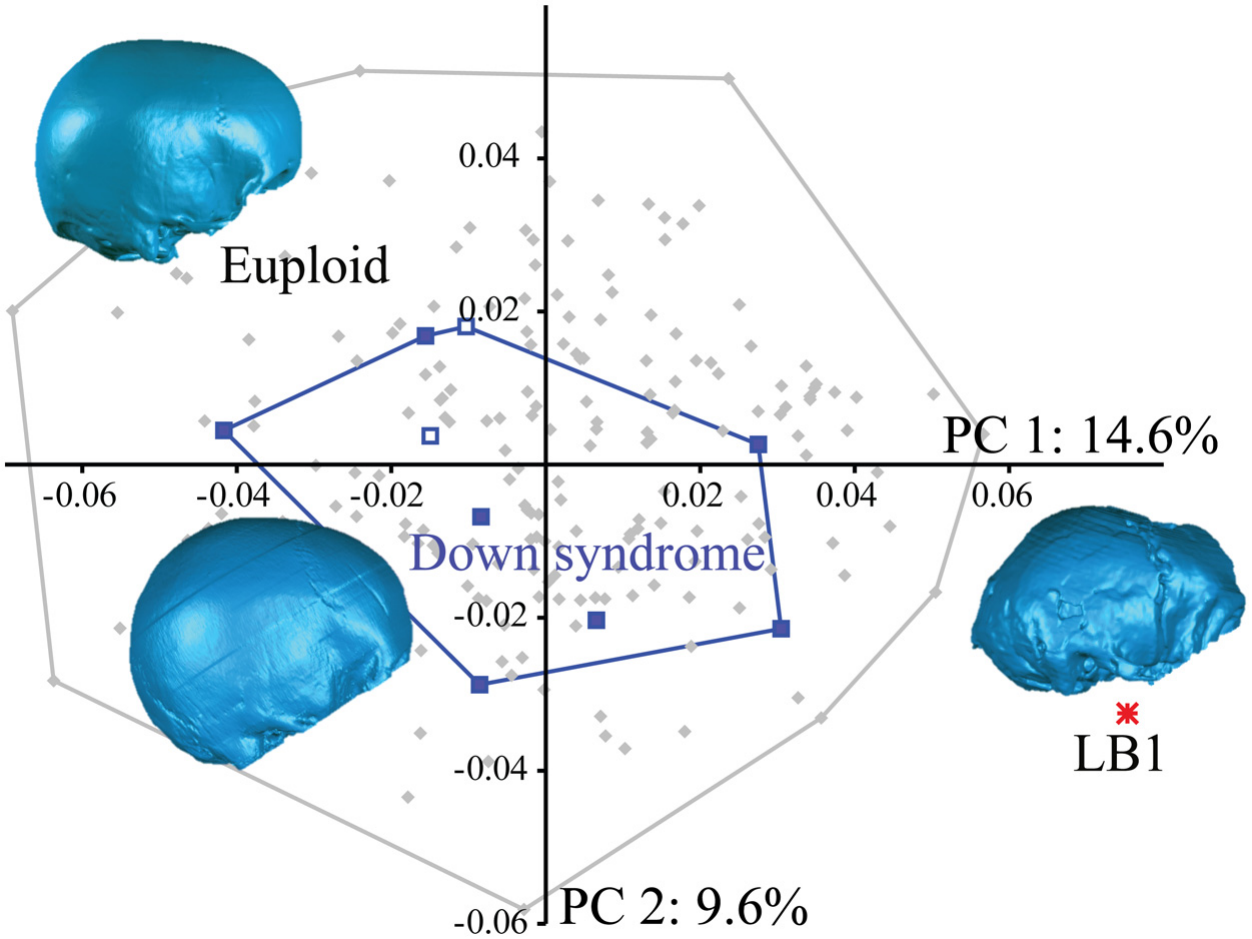
Principal components 1 and 2 of a PCA of neurocranial shape based on 3D landmarks (see Materials and Methods) in euploid and DS samples of humans and LB1. The LB1 neurocranial shape is distinct from the two modern human samples, which themselves evince considerable overlap in shape. The solid blue squares are the adults with DS, the blue outlined squares are juvenile / subadults with DS, gray diamonds are euploid adults and the red asterisk is LB1. Surface renderings are single examples from each group and are for illustrative purposes only. The first two components accounted for 14.6% and 9.6% of the total variance, respectively (PC 1 eigenvalue = 0.0006; PC 2 eigenvalue = 0.0004).

Henneberg, Eckhardt [25] focused on the brachycephalic index and low cranial profile of LB1 as support for a DS classification. Brachycephaly in individuals with DS appears to be due to a proportionally greater reduction in antero-posterior growth of the vault relative to medio-lateral growth [49, 78]. Lestrel and Roche [78] found that this disproportionate reduction led to a relatively higher vault in DS than euploid individuals, which contrasts with the low vault of LB1. We confirm this using quantitative analysis of 3D landmarks capturing cranial vault shape which indicates that the low neurocranial profile of LB1 was distinct from both euploid and DS modern humans on PCs 1 and 4, who themselves overlapped to a large extent (Fig 3). The landmarks that loaded most strongly on PC 1 included many of the midline landmarks (e.g., lambda, bregma and inion), but also frontotemporale (due to greater postorbital constriction in LB1), and landmarks such as asterion and anterior pterion that reflect the differences in relative breadths of the vault (LB1 is relatively wider inferiorly and narrower anteriorly). Neurocranial size (as captured by centroid size) accounts for only a small proportion of variation in PC 1 scores (*R*^*2*^ = 0.05; *p*<0.05). Excluding LB1 from the regression actually strengthens the relationship slightly (*R*^*2*^ = 0.10; *p*<0.05) because LB1 scored much higher than predicted by its size based on the scaling relationship within the human sample. The relationship between size and PC 4 scores was not statistically significant. The DS centroid was distinct from the euploid centroid on some higher components, but the two samples always evinced substantial overlap in their ranges, consistent with the relatively minor shape differences discussed by Kisling [49] and Seward et al. [79] for these groups. In this context, the observation that LB1 and most DS individuals (as well as some euploid individuals) evince brachycephaly is eclipsed by the overall incongruent cranial architecture of LB1 and all humans.

Flat cranial base: Numerous workers have documented the open cranial base angle (platybasia) of DS individuals compared with euploid controls [49, 58, 59, 80, 81]. Alio et al. [58] showed that postpubescent individuals with DS had an average value of 144.0° (4.83) compared with 135.6° (4.31) in controls, similar to what Suri et al [59] demonstrated in children, both using the standard orthodontic measurement for cranial base angle (CBA) of basion-sella-nasion. A value of 130° was reported for LB1 using the anthropological measure of CBA (basion-sella-foramen cecum) [1]. Given the more superior position of foramen cecum relative to nasion, the equivalent basion-sella-nasion angle would actually be even lower, placing LB1 ~3 SD below the average DS value reported in [58]. LB1 does not therefore have a flat cranial base.

#### Facial anatomy

**Fig 4:**
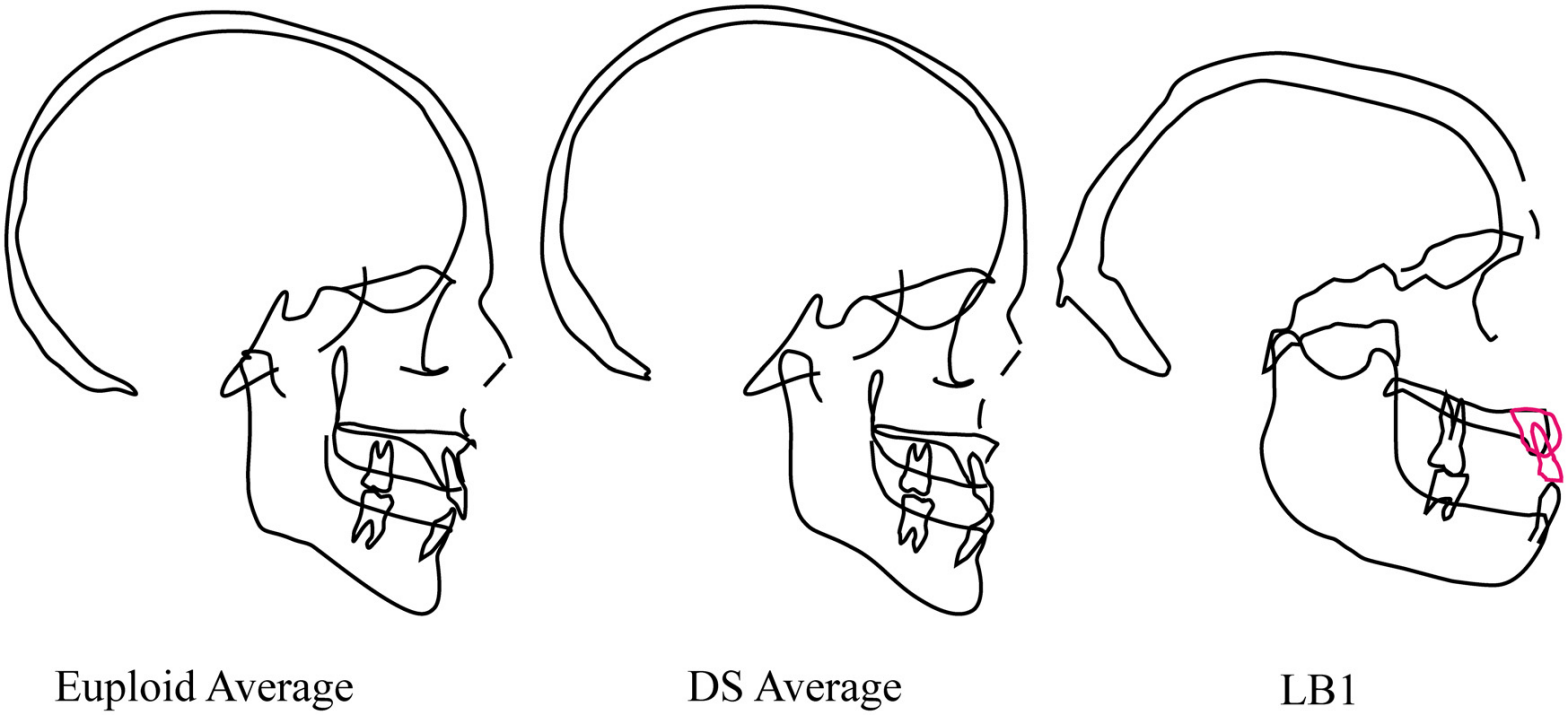
“Cranial templates” for euploid and DS samples of males between the ages of 19 and 29 years and pseudo-lateral cephalogram tracing of LB1. The euploid and DS cephalograms were based on average roentgencephalometric dimensions (modified from Kisling [49]). All three images have been scaled to approximately the same cranial length. Midfacial hypoplasia in the DS facial phenotype is apparent and contrasts strongly with the relatively long and prognathic maxilla and mandible of LB1. Other differences include the thicker cranial bones, shape of the mandible and the low neurocranial profile of LB1. Note that the LB1 cranium suffered damage to midline structures of the face, including the glabella, nasal bones and subnasal region; morphology of anterior maxilla was estimated based on surrounding morphology and indication of edge-to-edge occlusion of incisors by PB.

A small midface and maxilla are among the most characteristic features of the DS craniofacial phenotype (e.g., [82]) (Table 1). This configuration results in midfacial hypoplasia and malocclusion including mandibular overjet and anterior open bite, which are also affected by the protruded and proinclined lower incisors often seen in DS patients [49, 83, 84]. Henneberg et al. ([25], p. 4 of SI) did not list a disproportionately small midface and maxilla as skeletal manifestations of DS in LB1, but did describe the maxilla of LB1 as “underdeveloped” which they claimed contributes to “the reduced midfacial skeleton situated superior to a disproportionately underdeveloped mandible.” According to Spitzer et al. ([51], p. 569), “The diminutive maxilla remains retracted under the protruding forehead…” in DS. Although we were unable to quantify this morphology directly as most of our imaging datasets for DS patients were de-identified by removing the face and there was midline damage to the LB1 face, it is nevertheless visually apparent that this description is the reverse of the condition seen in LB1 where the forehead is posteriorly sloping and the mid- and lower face are protruding (Fig 4). Moreover, the wear pattern on the lower incisors of LB1 that indicates edge-to-edge occlusion of the incisors [8] is likewise inconsistent with mandibular overjet, anterior open bite, or an “underdeveloped mandible.” The images in Fig 4 further suggest that the LB1 mandible is not underdeveloped but rather disproportionately large relative to cranial length by comparison to the DS and euploid human groups (e.g., ramus breadth).

Henneberg et al. [25] pointed to a high level of asymmetry in the craniofacial skeleton of LB1 as consistent with higher levels of fluctuating asymmetry recorded for soft tissue facial features in a sample of DS individuals as further support for a DS diagnosis. These two types of asymmetry represent varying descriptions of identifiable patterns asymmetry variation discussed more fully in S1 Text.

#### Symphyseal anatomy

Henneberg, Eckhardt [25] listed “Microgenia (Micrognathia)” as a clinical sign of DS seen in LB1 and LB6, citing Brown and Maeda [8]. Micrognathia refers to the underdevelopment of the mandible and is rarely listed as a feature of DS, although it is found in other chromosomal disorders (e.g., trisomy 13). Microgenia refers to a small, receding or “weak” chin where the soft tissue landmark pogonion does not project as far anteriorly as the vermillion border of the lower lip. Microgenia cannot therefore be evaluated in LB1 in the absence of soft tissue anatomy.

**Fig 5:**
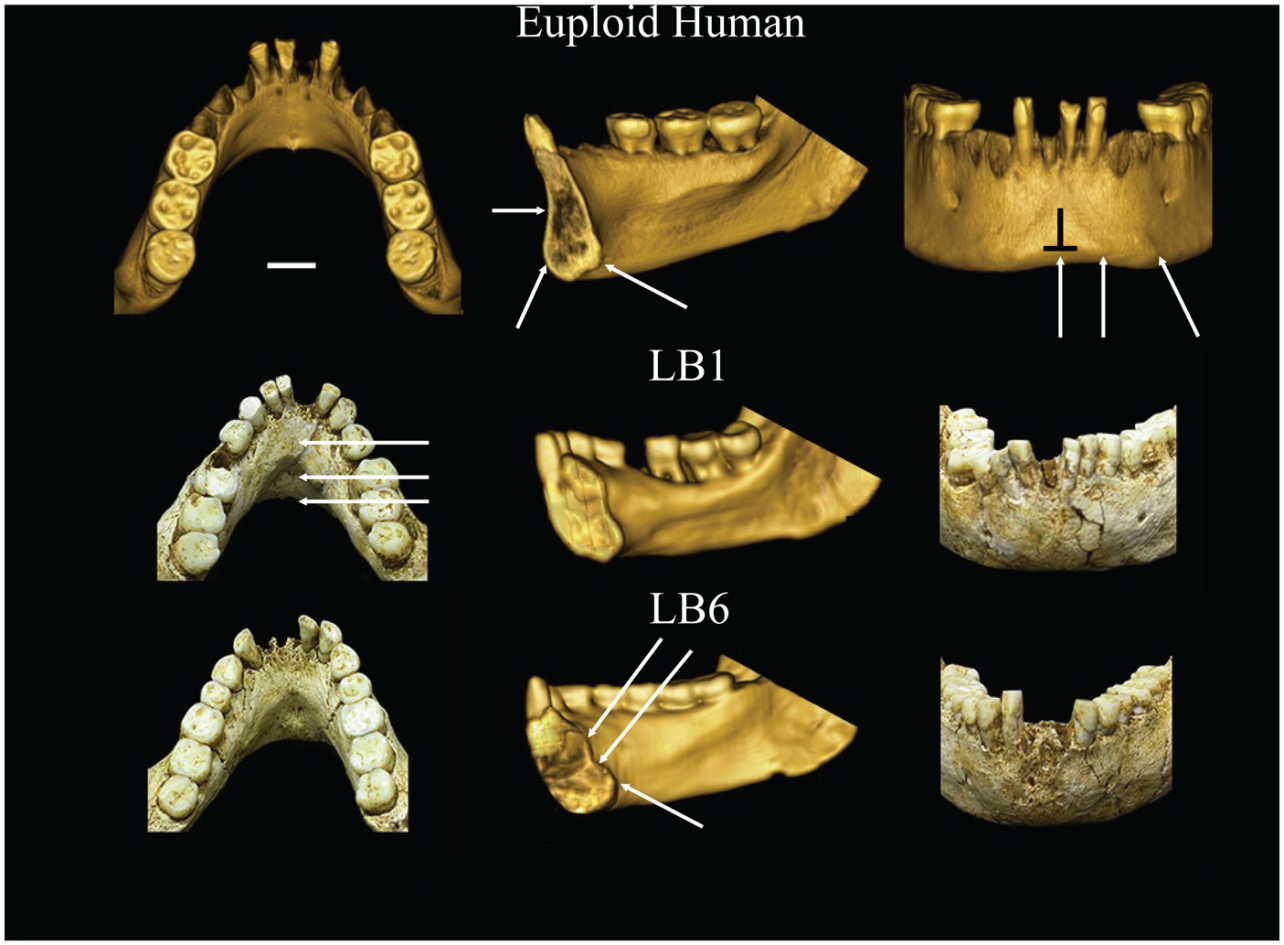
Comparison of symphyseal anatomy, shape and dimensions in a euploid modern human, LB1 and LB6. Note presence of chin (mental protuberance or trigone), inverted T, incurvature and absence of internal buttressing in modern human. LB1 and LB6 are similar anatomically and distinct from *H*. *sapiens*. No mental protuberance, incurvature, inverted-T, or tubercles. LB1 and LB6 have inferior and superior transverse tori, with deep genioglossal pit.

**Fig 6:**
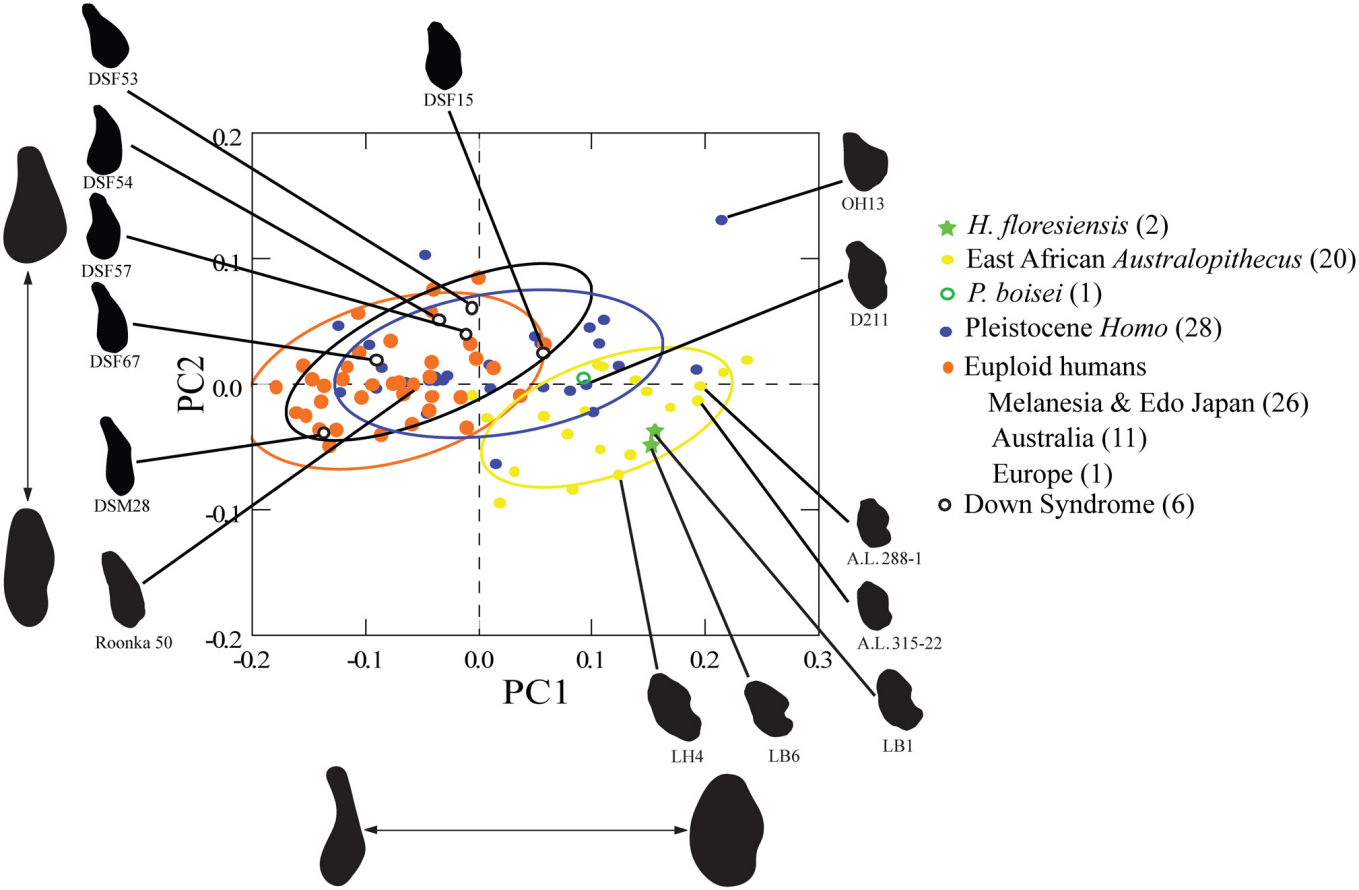
Principal components 1 and 2 of a PCA of symphyseal shape based on Fourier shape variables. Shape differences (anterior facing left) associated with the PCs are illustrated below (PC 1) and to the left (PC 2) of the ordination. The samples include regionally appropriate modern humans, individuals with DS, Pleistocene *Homo*, *Australopithecus* and one *Paranthropus boisei* fossil, as well as LB1 and LB6. LB1 and LB6 are quite similar in their symphyseal shape and most closely resemble *A*. *afarensis*. The DS sample overlaps the euploid modern humans and some Pleistocene *Homo* samples. The first two components accounted for 73.1% and 9.6% of the total variance, respectively (PC 1 eigenvalue = 0.01; PC 2 eigenvalue = 0.001).

However, other aspects of symphyseal anatomy can be assessed. The overall anatomical configuration presented by LB1 and LB6, consisting of a smooth and receding anterior symphysis (no boney hallmarks of a chin), and superior and inferior transverse tori separated by a deep/broad genioglossal fossa posteriorly, aligned them with archaic hominins, including australopiths, rather than modern humans (Fig 5) [1, 8]. This was further borne out by a PCA of symphyseal cross-sectional shape (quantified by elliptic Fourier analysis) demonstrating that LB1 and LB6 did not overlap the DS or euploid samples and are most similar to one another and to *Australopithecus afarensis* (Fig 6). The major shape differences between the DS (and euploid sample) and LB1 / LB6 (and australopiths) include the thicker (antero-posteriorly expanded) symphysis with a smooth, bulging anterior contour and a double projection on the posterior border corresponding to the superior and inferior transverse tori in the latter.

#### Cranial sinuses

Another common clinical finding in DS is small or absent cranial sinuses [49, 51, 85–87]. In particular, frontal sinuses are usually congenitally absent (83–93%; [50, 51, 86]) or more rarely hypoplastic (3.4%; [50]). Maxillary sinuses are sometimes poorly developed (6.9–40%; [50, 53]). Sphenoid, mastoid and ethmoid sinuses have been described as poorly developed or poorly pneumatized [51, 88]. Specifically, Spitzer et al. [51] found the sphenoid sinus to be restricted to the anterior sphenoid bone in ~66.6% of DS cases they examined, while Miller et al. [52] found that 20% and 40% of DS patients aged 16–60 had non-pneumatized or partially pneumatized sphenoid sinuses, respectively.

A frontal air sinus previously identified in the right supraorbital torus based on clinical CT data [19, 20], was reinterpreted as a defect or alteration in the diploë based on micro-CT data [89]. Frontal sinus development is difficult to assess in the area around glabella due to excavator damage, but Balzeau and Charlier [89] did not observe pneumatization in the surrounding region and viewed this as evidence for absence of the frontal sinus.

**Fig 7:**
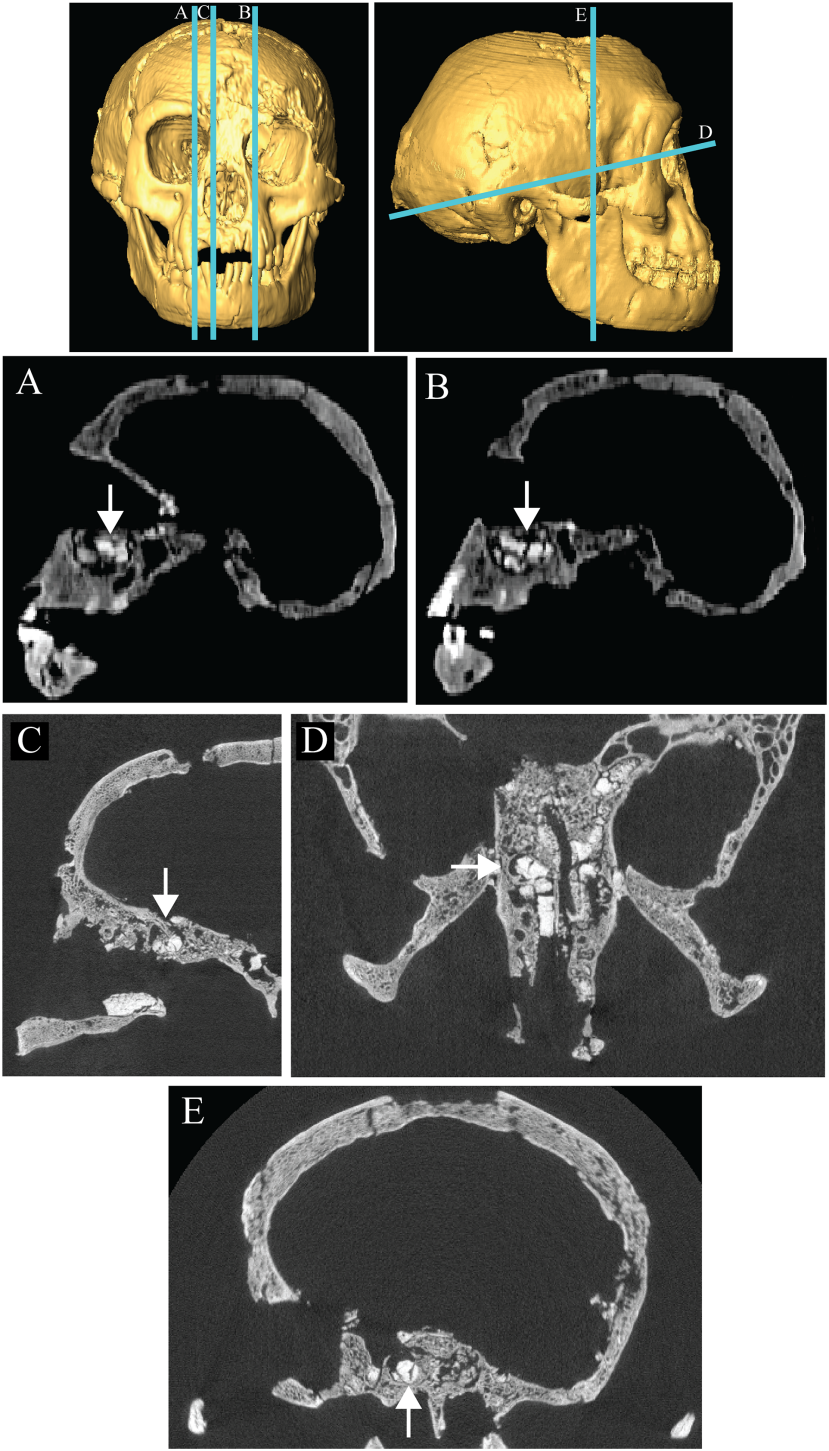
Matrix-filled maxillary and sphenoid sinuses in LB1. Arrows indicate the right (A) and left (B) maxillary sinuses as seen in parasagittal sections of the LB1 skull using medical CT imaging. The probable sphenoid sinus is illustrated in parasagittal (C), transverse (D) and coronal (E) sections based on higher resolution micro-CT scans of the cranium. The positions of the three sections are shown on the surface renderings in the top row. Micro-CT images (C-E) were provided courtesy of Yousuke Kaifu.

Henneberg, Eckhardt [25] expressed doubt regarding the presence of a sphenoid sinus in LB1 and asserted that there was little space for the development of a maxillary sinus in the vertically short and “sunken midfacial (infraorbital) regions” (p. 4 of SI). We provide here additional images from medical CT scans that show the left and right maxillary sinuses in LB1 (Fig 7). Although they are filled with matrix, this is of a different density than the surrounding bone [19]. The anatomy of the sphenoid body is more difficult to interpret definitively using the medical CT images alone. However, a higher resolution dataset based on a micro-CT scan of the cranium (see [3]) suggests the presence of a matrix-filled sinus (Fig 7) which is more visible on the right side in this particular image, but can also be identified on the left side. Aplasia of the sphenoid sinus is therefore deemed unlikely. The sinus may be restricted to the anterior part of the sphenoid body, but it is difficult to identify its borders with sufficient confidence to either support or refute the possibility of sphenoid sinus hypoplasia. Mastoid air cells are well-developed in LB1 (see Fig 3 in [20]).

#### Cranial vault thickness

Another possible skeletal sign of DS is thin cranial bones ([51, 85], but see [90]). LB1’s cranial bones are not thin by the standards of modern humans (see Fig 7 here, and Fig 3 in [20]). Absolute values for cranial vault thickness in LB1 are similar to or above the averages for a global sample of euploid humans and similar to or lower than average values for *H*. *erectus* [1, 3, 19, 89]. A similar pattern applied when only Holocene aboriginal Australians were considered[19]. Although some locations on the frontal bone are thicker in LB1 than in euploid humans, this may be artificially increased in LB1 by the hyperostosis frontalis interna documented by Balzeau and Charlier [89].

Cranial bone thickness may scale with body mass and endocranial volume in hominins [91, 92]. Lestrel and Roche [93] demonstrated that cranial bone of adults with DS was thinner than euploid adults terms of both absolute and relative thickness (standardized by area). The cranial bones of LB1 are likely thick relative to size compared to euploid humans given their overlap in absolute values and the small EV of LB1, but this has not been evaluated quantitatively. The absolute values for cranial vault thickness in LB1 cannot be characterized as thin compared to euploid humans, including regional samples (contra [94]), and are likely to be relatively thick when the small EV of LB1 is taken into account (see also [89]).

#### Dentition

The dental clinical literature describes a number of traits that are more common in DS patients than unaffected sections of the population. These include, relatively small teeth, hypodontia, oligodontia, tooth transposition, agenesis, occlusal abnormalities, and a relatively small dental arch (S1 Table). While none of these traits are pathognomonic signs for DS, a combination of these traits might form the basis for a more convincing differential diagnosis than provided by [25]. Henneberg et al. [25] mention irregular position of teeth and missing teeth in LB1 as consistent with DS but do not provide additional details.

LB1 and LB6 do not have relatively small teeth or palates. For example the medio-lateral and bucco-lingual dimensions of the mandibular molars of LB1 and LB6 are quite similar to those of a large sample of modern humans, and the LB1 molars are actually slightly larger than those of LB6 [8]. Edge-to-edge anterior tooth wear in LB1 and LB6 is not consistent with open bite or mandibular overjet, and there are not any transposed teeth [8, 95]. The maxillary P^2^s are bilaterally rotated 90° in LB1 [95]. Unilaterally, these are the most commonly rotated teeth in modern humans [96], but there are no known syndromic associations [97]. Additional discussion can be found in S1 Text and illustration of the non-taurodont M_1_ of LB6 is found in S2 Fig.

Hypodontia (missing up to 5 teeth, excluding M3’s) and oligodontia (missing >5 teeth, excluding M3’s), with teeth failing to develop, are widely reported features of DS [54]. Most frequently absent teeth in DS are third molars (74%), lateral incisors (I^2^ >26%; I_2_ >20%) and 2^nd^ premolars (P^4^ >18%; P_4_ >25%) [98–106], which are the same teeth most often missing in the general population [107]. Two teeth, the right P_4_ and right M^3^, were not present before death in LB1, with no evidence of their dental alveoli [95]. Whether the right P_4_ was congenitally absent, or lost during life is uncertain. There is a space between the neighboring P_3_ and M_1_ where the P_4_ would have been, and interproximal facets on the distal P_3_ and mesial M_1_. However, these observations are also consistent with a retained deciduous molar (see also [108]). However, if the P_4_ failed to develop, then the dm_1_ should have been retained, or mesial migration reduced the space between P_3_ and M_1_ more than it has [109, 110], unless this occurred soon before death. There is no evidence of the P_4_ being lost due to caries as speculated by [111]. Remaining sections of posterior alveolus, and mandibular incisor wear, indicate that LB1 had a complete set of maxillary incisors. The incisors were lost as part of the excavation damage to the face and left frontal region in 2003 [8, 95]. Tooth wear, with no distal facet on the right M^2^, indicates agenesis of the M^3^. LB6 presents the full complement of mandibular teeth.

### Stature and Postcranial Skeleton

#### Stature

LB1’s extremely short stature was an outlier even with respect to the reduced statures of people with DS. Rounded to the nearest cm, our range of statures estimated for LB1 from the femur and femur+tibia was bracketed between 1.00–1.09 m (Table 3), values which were comparable to the original estimates of 1.04–1.09 m based on the femur only [1]. Our tibia+femur estimates were encompassed within the larger range of estimates from the femur only. Classic calibration works best when extrapolation is required to predict stature, as is the case here [112]. For this reason, we suggest that while 1.00–1.09 m represents a reasonable stature range for LB1, we prefer the lower values from classic calibration (1.00–1.04 m) because extreme extrapolation is required even for the pygmy training sample. Application of the Konigsberg et al. [112] classic regression for the femur from a large sample of normal sized modern humans yields an estimate of 1.08 m for LB1, a value consistent with our new results from pygmies. Henneberg, Eckhardt [25] presented a range of stature estimates for LB1 based on several reference populations and both upper and lower limb elements. Use of the upper limb elements is ill-advised in view of LB1’s interlimb proportions being outside the range of modern humans of any stature [6]. Restricting the Henneberg, Eckhardt [25] estimates to those based on Australian and Asian reference samples, and utilizing only the femur or the tibia+femur, the range was 1.06–1.20 m (for females / mixed sex) and 1.33 m (for males).

**Table 3:** Stature estimates for *Homo floresiensis* using an African pygmy reference sample (N = 19).

	**Femur**	
	Inverse calibration (OLS)^1^	Classic calibration^2^
LB1 (280 mm)	108.6	100
	**Femur+Tibia**	
	Inverse calibration (OLS)^3^	Classic calibration^4^
LB1 (515 mm)	108.8	104.2
LB8^5^ (~473 mm)	101.5	95.8

^1^ Stature (cm) = [0.331 x femur length (mm)] + 15.876; r = 0.89, s.e.e. = 3.7

^2^ Stature = (femur length– 41.706)/2.3837

^3^ Stature = [0.173 x (femur length + tibia length)] + 19.694; *r* = 0.93, s.e.e. = 2.9

^4^ Stature = (femur length + tibia length + 6.6663)/5.0055

^5^ Femur length for LB8 is estimated from its tibia length (216 mm) and the crural index of LB1 (83.9)

**Fig 8:**
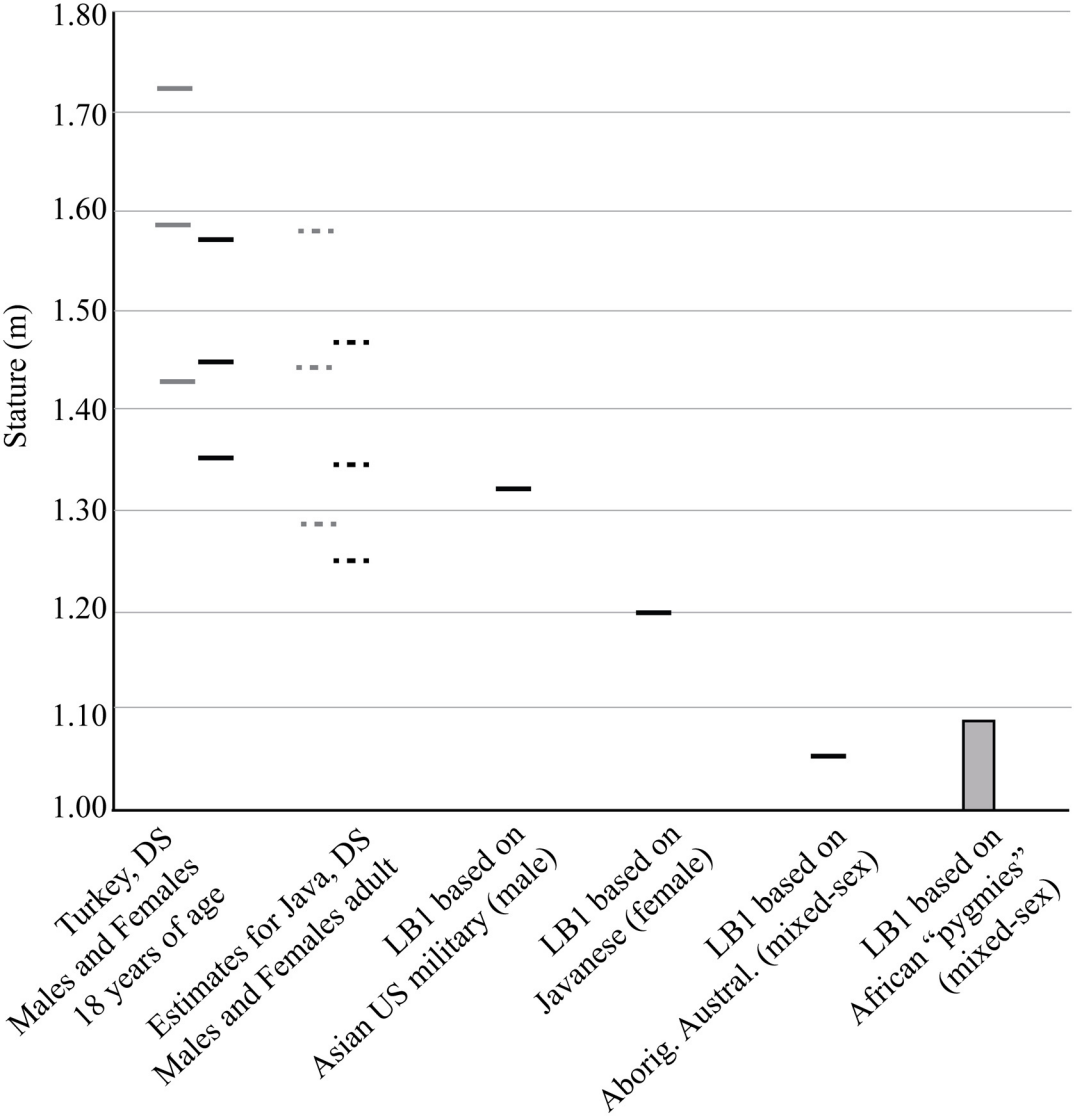
Observed stature for 18-year-old males (gray) and females (black) with DS from Turkey, comparable estimates for Javanese with DS, and estimates for LB1 based on different reference populations. The Turkish data are from Tüysüz et al. (2012). Estimates for Javanese with DS are based on the relationship between euploid and DS Turkish populations and average stature for adult male and female (euploid) Javanese (see text for details). The three lines indicate the 97^th^, 50^th^ and 3^rd^ percentiles. The first three stature estimates for LB1 are from Henneberg et al. (2014) while the range on the left were generated for this study.

LB1 stature estimates based on Javanese females (see [25] for sample details), a mixed-sex sample of Aboriginal Australians (see [25] for sample details), and small-bodied African populations (see [113] for sample details) were all well below the 3^rd^ percentile of stature for both Turkish (n = 1726; [114]) and estimated Javanese DS groups (see Methods for details) (Fig 8). Only the estimates for LB1 stature based on US military males of Asian descent (not the most appropriate reference population) provided by Henneberg, Eckhardt [25] were within the ranges of Javanese DS heights. LB8 would be an even more extreme outlier as the stature estimates from its femur+tibia ranged from 0.96 m (classic) to 1.02 m (inverse). A stature of ~1 m is achieved in Turkish females with DS at roughly 6 years of age.

#### Foot:femur ratio

**Fig 9:**
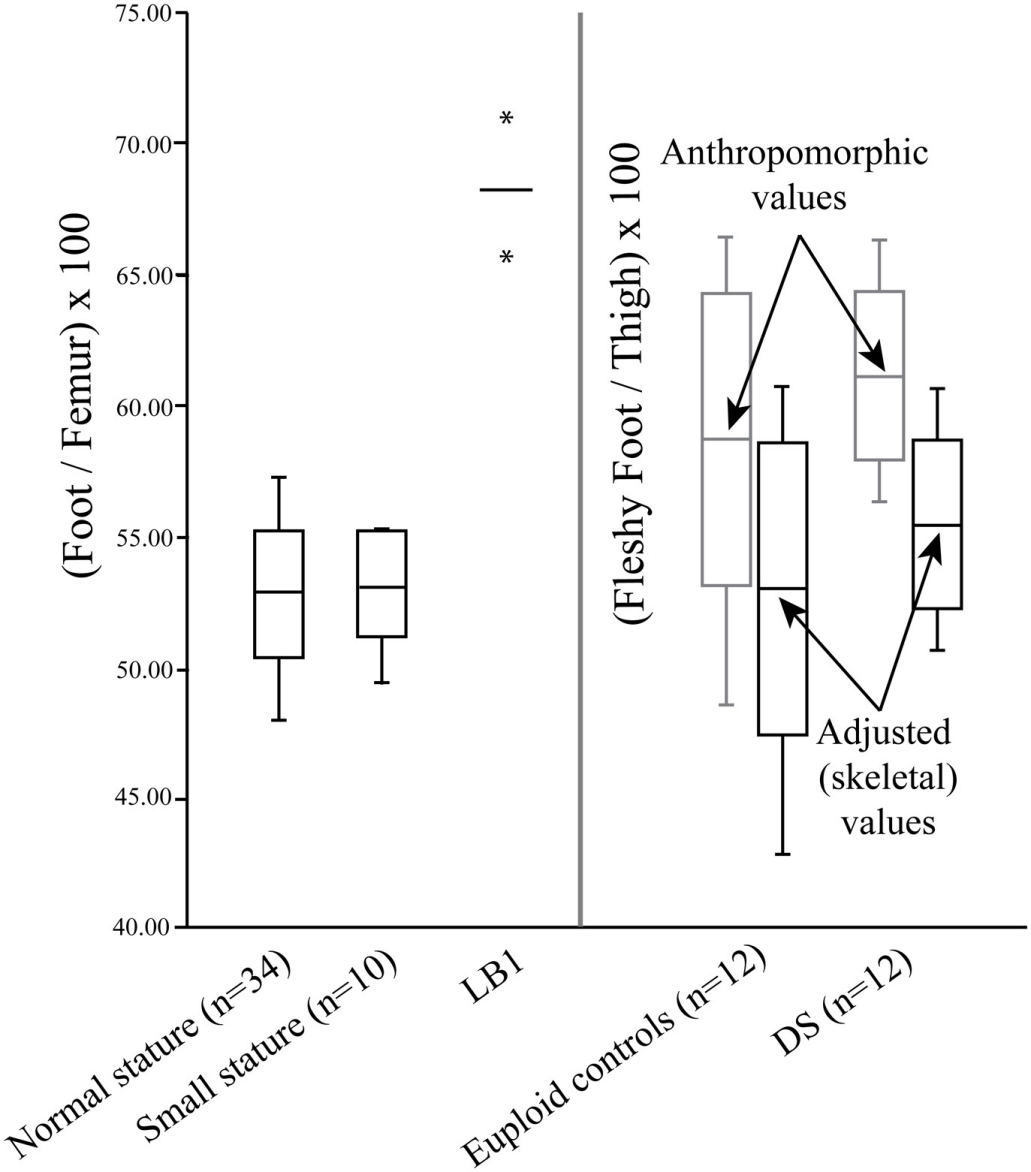
Plot of the foot:femur (or foot:thigh) ratio in recent humans of normal stature and short stature, LB1, and a DS and a matched euploid control sample. The box-and-whisker plots include the mean and ±1 SD as well as the range. The asterisks for the LB1 value are the 95% confidence intervals based on the regression equation used to obtain the estimate [7]. The fleshy foot:thigh bars labeled “anthropometric” are the values based on the data from the DS and euploid control samples from Smith and Ulrich [115], while the bars labeled “adjusted” represent the raw values adjusted to make them more comparable to the skeletal ratios (see text for details).

Henneberg, Eckhardt [25] argued that the short femur relative to foot length was a feature that linked LB1 to DS. They cited anthropometric data from Smith and Ulrich [115] that included mean lengths for the foot and thigh in DS (n = 12) and matched euploid samples (n = 12) in support of a higher femur:foot length ratio for individuals with DS. The DS ratio (0.61 unadjusted; 0.56 adjusted for comparability to skeletal ratios; see Methods for details) was on average slightly higher than the euploid values (0.58 unadjusted; 0.53 adjusted). They concluded that since the skeletal ratio of foot:femur for LB1 of 0.68 was closer to the DS value, this aligned LB1 with the DS sample. However, the slightly higher values for the DS versus euploid groups in no way approach the extremely high value of LB1 (Fig 9). The DS mean is within one standard deviation of the corresponding euploid value, and the DS range is entirely within the euploid range. On the other hand, the observation that LB1’s ratio is closer to the DS than the euploid average is of no importance given how extreme the LB1 value is relative to the euploid and DS values. This is confirmed by a *t-*test indicating that the LB1 value was significant higher than the DS average (*t-*value: 1.81, *p*: <0.001). Comparable data regarding the femur:humerus (thigh:upper arm) ratio are discussed in S1 Text and illustrated (S3 Fig).

#### Digit length

The hands of DS individuals are frequently characterized as broad and short (e.g., [35, 116]), but this description is unaccompanied by any metric quantification. The fifth finger in particular is short due to brachymesophalangia (short middle phalanx) [117], but this may be more relevant for young individuals than adults [45, 46]. Henneberg, Eckhardt [25] cite small dimensions for the Liang Bua manual and pedal phalanges, particularly the distal phalanges, relative to female European averages. As we are unaware of short toes (i.e., pedal phalanges) being part of the DS phenotype, only the manual phalanges were assessed. The absolute lengths of the proximal and intermediate manual phalanges of LB1, LB6 and LB10 (see Material and Methods for reassignment of LB10 first proximal phalanx from the foot to the hand) fell within the range of normal- and small-statured samples (Table 4). One of LB1’s distal phalanges was within the range of the human samples, but its pollical distal phalanx and the distal phalanges of LB6 were smaller than in our samples. However, the stature estimates for the Liang Bua individuals indicate that they are smaller than most modern humans, and therefore relative measures are more appropriate. Relative to humeral length, the pollical distal phalanx of LB1 was just below the range for the small-bodied groups (Table 4), but within the range for the normal-statured sample (S2 Table). Only ten individuals from the small-bodied sample preserved the first distal phalanx, so the true population range may in fact encompass this value. Overall, only the distal phalanges are small and even then they overlap or approach the modern human range when scaled by humeral length.

**Table 4:** Absolute and relative length of manual phalanges in the Liang Bua hominins and a comparative sample of small-bodied modern humans.

Manual Phalangeal Length				
Modern Humans				Liang Bua values
Digit	Average	SD	Range	
*Proximal*				Indeterminate digit—LB1:>33.4; LB6: 31.2
I	27.3	1.9	23.6–30.7	Digit I—LB10: 24.2
II	36.4	2.1	32.4–39.5	
III	41.0	2.6	37.0–45.2	
IV	37.9	2.0	34.2–41.9	
V	29.9	2.1	25.7–34.3	
*Intermediate*				Indeterminate digit—LB1: 25.6; LB6: 16.9
II	21.0	1.7	19.3–23.8	
III	25.4	2.1	21.6–29.8	
IV	24.6	1.9	22.1–28.6	
V	18.0	1.9	15.4–21.7	
*Distal*				Indeterminate digit—LB1: 13.4; LB6: (?)10.5–12.9
I	21.0	2.0	18.4–24.3	Digit I—LB1: 15.2
II	15.3	0.7	14.2–16.1	
III	16.4	1.5	14.9–19.5	
IV	16.5	1.9	14.7–19.8	
V	15.9	2.3	13.4–19.5	
Manual Phalangeal Length as % of Humeral Length (LB1 only)				
Modern Humans				Liang Bua values
Digit	Average	SD	Range	
*Proximal*				Indeterminate digit—>13.7%
I	9.9%	0.5%	8.8–10.8%	
II	13.1%	0.6%	11.7–14.2%	
III	14.8%	0.8%	13.4–16.1%	
IV	13.8%	0.5%	12.9–14.6%	
V	10.8%	60.0%	9.6–11.8%	
*Intermediate*				Indeterminate digit—10.5%
II	7.7%	0.4%	7.0–8.2%	
III	9.2%	0.5%	8.6–10.3%	
IV	8.9%	0.4%	8.4–9.7%	
V	6.4%	0.5%	5.8–7.5%	
*Distal*				Indeterminate digit—5.5%
I	7.6%	0.5%	6.9–8.6%	Digit I—6.3%
II	5.6%	0.3%	5.3–6.1%	
III	5.9%	0.4%	5.4–6.6%	
IV	6.0%	0.6%	5.3–6.7%	
V	5.7%	0.7%	4.9–6.7%	

Additional postcranial features discussed by Henneberg et al. [25], including craniocervical instability / hypermobility and flexible flat foot seen frequently in DS individuals are attributable to ligamentous laxity [118, 119] and are therefore difficult to assess in osteological remains. The abnormal boney anatomy of the atlanto-occipital joint in LB1 described by Kaifu, Baba [3] was interpreted by these same authors as indicative of **limited** mobility rather than hypermobility, and the “flat foot” described previously in LB1 reflects a primitive bony foot architecture, not flexible flat foot [7] (see S1 Text for more details).

Plagiocephaly and hypothyroidism were also listed as clinical signs in LB1 that sometimes occur in DS [25]. Kaifu, Baba [120] argued persuasively for the presence of positional (deformational) plagiocephaly in LB1 based on the particular pattern of asymmetry in the cranium and the pattern of occlusion. Although having DS is a risk factor for positional plagiocephaly, its presence in LB1 is not particularly strong evidence of DS given that <1% of individuals with positional plagiocephaly had DS in a large study of risk factors for positional plagiocephaly [121]. Reported frequencies of congenital hypothyroidism vary from 0.7–23.5% of newborns with DS [68, 69], but are considerably lower in children and adults (Table 1). It has been suggested that LB1 and other Liang Bua hominins had congenital (myxoedematous endemic) hypothyroidism (“cretinism”) resulting from a lack of dietary iodine [13–15]. While phenotypic features of congenital hypothyroidism resulting from lack of iodine or other causes (e.g., genetic) are likely to be similar, there is also considerable evidence indicating that the Liang Bua hominins did not have hypothyroidism [19, 21] and this cannot be considered strong evidence of a DS diagnosis for LB1.

## Discussion

Although a concrete diagnosis of DS can only be accomplished through genetic testing, we critically evaluated whether the skeletal anatomy of LB1 is compatible with the DS phenotype as proposed by Henneberg et al. [25]. We generated novel data sets related to endocranial volume, neurocranial and symphyseal shape in DS, as well as new stature estimations for LB1. We were unable to verify more than marginal overlap between the LB1 and DS phenotypes. In several instances, Henneberg et al. [25] described morphologies in LB1 that superficially resembled features seen in DS, but actually reflected different underlying phenotypes, including “atlanto-occipital abnormality” and flat feet. In fact, neither of these features were directly evaluable in LB1 as they relate to soft tissue structures, as is also the case for microgenia. In other cases, qualitative similarity was not borne out by quantitative analyses, such as small brain, short stature, brachycephaly, and short femora. In the case of facial asymmetry, Henneberg et al. [25] conflated previous measures of fluctuating asymmetry of the face (soft tissues) in a sample of people with DS with asymmetry of the LB1 facial skeleton (discussed in S1 Text). There were a small number of cases where the results were more ambiguous and require careful consideration, such as short manual digits, aplastic / hypoplastic cranial sinuses and tooth agenesis.

The clinical literature suggests that some or many DS individuals are characterized by a slightly smaller than average brain size (and accompanying head circumference). The cranium is often described as brachycephalic with a flat cranial base, a hypoplastic midface and disproportionately small maxilla. The mandible is usually of normal size but individuals with DS may present with microgenia. DS individuals are further characterized by reduced stature and higher foot:femur or fleshy foot:thigh ratios than euploid control groups.

In contrast, the greatly reduced brain size (EV = ~417 cm^3^) and head circumference of LB1 mark this individual as an outlier relative to those DS individuals evaluated here whose values are much closer to control samples of euploid individuals. Similarly, the low and poorly filled out cranial vault of LB1 contrasts sharply with samples of both DS and euploid modern humans who are themselves quite similar, and is more similar to *H*. *erectus* [21]. The cranial base of LB1 is not flat, and its cranial base angle overlaps the euploid but not the DS range of values. The anteriorly projecting face and posteriorly sloping frontal squama of LB1 do not match descriptions of DS individuals but are common in archaic *Homo*. The posteriorly sloping anterior symphysis of the mandible lacks the elements of a bony chin present in euploid and DS samples, and exhibits both superior and inferior tori on its posterior surface, features that align it more clearly with early hominins than with either DS or euploid humans. The short stature (just over 1 m) and disproportionately short lower limbs reveal further evidence of a primitive body shape that does not overlap ranges of variation for either DS or euploid samples. These examples confirm that the LB1 phenotype (and that of other Liang Bua fossils assigned to *H*. *floresiensis*) is better interpreted as a distinct species whose affinities lie with early *Homo* species than as a member of *H*. *sapiens* with DS or any other pathology proposed thus far.

LB1’s cranial morphology can be compared to descriptions and images of crania previously diagnosed with DS in the archaeological record from England (a Saxon burial) [44] and France [42]. Both Brothwell [44] and Rivollat et al. [42] cited brachycephaly, a flat posterior vault / occiput, thin cranial bones, reduced facial height and dental anomalies in support of their DS diagnoses. This brachycephaly is not accompanied by a dramatic decrease in cranial height nor by a posterior sloping of the forehead [44], both features that distinguish LB1 from the Saxon and French juvenile crania. Moreover, the strongly flexed occipital bone with a transverse torus and thick cranial bones in LB1 contrast with these DS diagnoses (S1 Table) [1, 8]. That said, we do not expect that all individuals with DS would present identical morphologies given that DS is highly heterogenous [36]. Indeed, the Saxon and French juveniles differ from one another in features such as cranial base flexion and microcephaly and some features in each more closely resemble the euploid than the DS condition. However, in both cases there are numerous craniodental characteristics that frequently differentiate DS from euploid individuals, which is not the case for LB1.

In the case of short manual digits, we found that the distal phalanges of the Liang Bua hominins were short by modern human standards but in most cases those of LB1 were within the modern human range, particularly when scaled by humeral length. As we are uncertain what digit most phalanges belong to, we cannot conclusively state whether they are long or short with comparison to modern human populations. The lack of quantitative data on DS digit length further complicates interpretation of this pattern. However, it is particularly notable that LB6, a second individual from Liang Bua cave that was not diagnosed with DS, had even shorter distal phalanges than LB1. This suggests that this is a population characteristic rather than an abnormal condition in LB1.

The issue of small or missing paranasal sinuses remains only partially resolved. The most commonly missing sinus in DS is the frontal sinus. A centrally positioned frontal sinus cannot be directly evaluated due to excavator damage in the median plane of LB1, but Balzeau and Charlier [89] found no evidence of pneumatization in the vicinity using micro-CT data. The less damaged right supraorbital torus was previously described as containing a frontal sinus [19, 20], but this was questioned by Balzeau and Charlier [89] using their higher resolution dataset. Frontal sinus aplasia cannot therefore be ruled out. Maxillary sinuses are present (ruling out aplasia) and do not appear especially small relative to overall facial size, also excluding a diagnosis of Type III maxillary sinus hypoplasia (cleft-like). Establishing the presence of Type I or II maxillary sinus hypoplasia would require evaluation of delicate internal structures such as the uncinate process and infundibular passage, as well as soft tissue opacification, neither of which can be assessed currently [122]. There appears to be a sphenoid sinus, but its boundaries and position relative to the sella turcica (the landmark used most commonly in the clinical literature) are challenging to clearly identify. Taken together, evidence from the cranial sinuses neither strongly supports nor refutes a DS diagnosis for LB1.

We provided vital contextual information about the distribution of periodontal disease and dental caries. This context indicates that the presence of periodontal disease and lack of caries in LB1 is not uncommon in archaic hominins or modern hunter-gatherer populations, and, in the absence of a host of other common DS dental anomalies, is not convincing evidence of DS. We also evaluated a number of dental features common in DS not seen in LB1, such as microdontia, tooth transposition, mandibular and maxillary incisor protrusion, and Angle’s class III malocclusion. We were unable to confirm claims that LB1 presented with taurdontism as do some DS individuals, but could not fully evaluate this feature with available radiographs.

Failure of permanent teeth to develop is a common finding in individuals with DS, and LB1 is missing a right P_4_ and M^3^. It remains unclear whether the P_4_ failed to develop or was lost during life. More than one third molar was affected in nearly all cases of third molar agenesis in DS (95%) documented by Shapira et al [123] in contrast to the single tooth missing in LB1, and Suri et al. [54] found that an average of 4.74 teeth were missing in DS patients. Moreover, the third molar is the most common tooth absent due to agenesis in the general population, with ~30% of some Asian populations exhibiting M^3^/M_3_ agenesis [124]. Likely instances of third molar absence have also been documented in fossil hominins, including early *H*. *erectus* [125–127]. Hypodontia affects between 2.8–11.3% of the general population, and the same teeth are most often involved as in DS [107, 124, 128].

DS is a phenotypically variable disorder and its clinical signs are neither unique to this disorder nor universally expressed in all individuals with DS. Hence, it is not necessary for LB1 to exhibit all hard tissue features that are consistent in modern individuals with DS to posit the presence of this chromosomal disorder. Yet, the presence of only a handful of features is insufficient to confirm a differential diagnosis of DS. Of the clinical signs associated with DS, only short distal phalanges, M^3^ agenesis, possibly hypodontia and possibly hypoplasia of one or more cranial sinuses was consistent with this diagnosis. This list of features are not unique to DS and are not among the most diagnostic (e.g., midfacial hypoplasia). This, combined with the large number of features common to the DS phenotype that were not present in LB1, indicates that it is highly unlikely that this individual had Trisomy 21 or DS. LB1 remains the type specimen of *H*. *floresiensis*, a species with its roots in Plio-Pleistocene *Homo*.

## Material and Methods

### CT and MRI data of DS patients

Computed tomographic (CT) and magnetic resonance imaging (MRI) data on 28 patients with a clinical (or karyotypic) diagnosis of DS and without other abnormal morphologies were acquired from Barnes-Jewish-Christian Hospital in St. Louis, Missouri (CT data) and from Richard Haier’s research group at University of California, Irvine (MRI). The Washington University School of Medicine IRB committee approved the analysis of de-identified CT data for this study (IRB ID #: 201410059: title "Down's syndrome and Hobbit"). The Midwestern University IRB committee determined that use of the MRI data did not meet the definition of human subject’s research and did not require additional approval. The latter images were originally acquired to identify indicators in the brain for Alzheimer’s in middle-aged adults with DS [129]. Surface renderings of the neurocranium were generated using the Mimics software package for subsequent landmark analysis. Additional details are provided in the S1 Text.

### Endocranial measurements

Virtual endocasts were created for the six DS females for whom CT data were available (age range: 10–67 years) following previously described methods [130], from which four linear measurements and endocranial volume (EV) were calculated. LB1’s value was compared with the mean value for our six subjects with DS for each measurement, and descriptive statistics were calculated. We also tested the null hypothesis that the LB1 value was not lower than the mean value for our 6 DS subjects (one-tailed test). Because six t tests were performed, to protect against Type I error, statistical levels of significance were corrected by adjusting for the false discovery rate [131]. In addition, we evaluated whether our small sample of 6 individuals with DS (the only one for which we had linear measurements) was representative of the DS population by means of a t-test comparing the EV of these 6 subjects to the 30 subjects with DS reported by Aylward, Habbak [73]. Images of virtual endocasts for six euploid females from Falk, Hildebolt [130], our six DS subjects, and LB1 were created and visually assessed. Statistical analyses were performed with JMP Pro Statistical Software Release 11.0.0 (SAS Institute, Inc., Cary, NC) and MedCalc Statistics for Biomedical Research Version 15.2.1.0 (MedCalc Software, Mariakerke, Belgium).

In addition, because of the small sample size, power calculations were performed with Power and Precision Release 4.1 (Biostat, Inc., Englewood, NJ). For all comparisons listed in Table 2, the statistical power exceeds 99.9%. More specifically, for the largest *P* value (0.009 for cerebral width), the null hypothesis was that the mean cerebral width for the DS population is 124.95 mm. With alpha (the criterion for significance) set at 0.01, a two tailed test (which means that an effect in either direction is interpreted), and our sample size of 6 subjects with DS, the power exceeds 99.9%. This computation assumes that the population from which the sample was drawn has a mean of 124.95 mm with a standard deviation of 5.76 mm. The observed value was tested against a theoretical value (constant) of 103.40 mm. Although we used a one-tailed test in our tests for differences, we used a two-tailed test for our power calculation (which results in less statistical power) and used a conservative alpha level of 0.01, which results in broad confidence intervals.

### Neurocranial shape

We quantified shape variation of the external neurocranium using a set of 39 3D landmarks (Table 5) acquired from dry skulls of geographically diverse adult euploid humans (n = 263) (see [132] for more details), surface renderings (from CT and MRI data) of humans with DS (n = 9), and LB1. Of the 9 individuals with DS, 7 were female and 2 were male. One female was a juvenile (10 years of age) and one was a subadult (15 years of age), while the remainder of the sample ranged from 35 to 54 years of age. Missing bilateral landmarks were estimated by mirroring its antimere (reflected relabeling) [133]. Landmark configurations were superimposed via generalized Procrustes analysis to remove the effects of scale, translation and rotation coded in the raw coordinate data [134, 135]. The x, y, and z coordinates of the 39 superimposed landmarks were then subjected to a principal components analysis (PCA) to reduce the dimensionality of the data and assess the main patterns of neurocranial shape variation. Operations were performed in SAS (SAS Institute, Inc., Cary, NC) and the PAST software package [136].

**Table 5:** Landmarks used in this study.

Landmark	Definition
Inion	Point at which superior nuchal lines merge in midsagittal plane
Lambda	The apex of the occipital bone at its junction with the parietals, in the midline
Bregma	Posterior border of the frontal bone in the midsagittal plane
Supraorbital notch	Point of greatest projection of notch into orbital space, taken on medial side of notch
Frontomalare temporale	Point where the fronto-zygomatic suture crosses the temporal line
Frontomalare orbitale	Point where the fronto-zygomatic suture crosses the inner orbital rim
Mid-torus inferior	Point on inferior margin of supraobrital torus roughly at the middle of the orbit (on superior margin of orbit)
Mid-torus superior	Point on superior aspect of supraorbital torus, directly above mid-torus inferior on anterior aspect of torus
Anterior pterion	Where coronal suture intersects spheno-frontal or spheno-parietal suture
Porion	Uppermost point on the margin of the external auditory meatus
Auriculare	Point vertically above the center of the external auditory meatus at the root of the zygomatic process
Frontotemporale	Point where the temporal line reaches its most antero-medial position on the frontal
Asterion	The common meeting point of the temporal, parietal, and occipital bones, on either side
Opisthion	Midline point at the posterior margin of the foramen magnum
Tympano-mastoid fissure	Point on lateral border of the tympano-mastoid fissure
Medial petrotympanic crest	Most medial point of petrotympanic crest at level of carotid canal
Lateral petrotympanic crest	Lateral origin of petrotympanic crest; if the petrotympanic crest splits, point is taken posteriorly
Postglenoid process	Infralateral-most point posterior to glenoid fossa and anterior to ectotympanic tube (postglenoid tuberosity or crest)
Inferior entoglenoid	Most inferior point on the entoglenoid pyramid
Temporo-sphenoid suture	Point where temporo-sphenoid suture passes from squama to cranial base (often on infratemporal crest)
Mid-parietal	Point on midsagittal suture midway between bregma and lambda (calculated from semilandmark data)
Mid-temporal	Point on the temporal squama midway between temporo-sphenoid and parietal notch (calculated from semilandmark data)

### Mandibular symphysis shape

The cross-sectional shape of the mandibular symphysis was quantified using elliptical Fourier analysis and analyzed using PCA as described in Brown and Maeda [8]. The comparative sample consisted of *Australopithecus* and Pleistocene *Homo* fossils, as well as one *Paranthropus boisei* fossil (KNM-ER 729) (Table 6), euploid humans from Melanesia, Edo Japan, Australia and Europe, six individuals with DS and the early Holocene Roonka 50 mandible discussed by Westaway, Durband [70] (see also [8]). The symphyseal sagittal cross sections for individuals with DS were obtained from lateral CT localizer radiographs (“scouts”) associated with the Barnes-Jewish-Christian Hospital series discussed above. Six of the localizer radiographs were from individuals without remodeling of alveolar bone associated with the loss of mandibular incisor teeth (age range: 15–67 y). Five of the individuals were the same ones used in the analysis of endocasts. Previous comparison of symphyseal cross-sectional shape outlines obtained from CT scan slices, lateral radiographs, sagittally sectioned mandibles and impression material did not indicate any significant differences in the resolution of shape and size [8]. We drew 68% confidence ellipses around each of the samples.

**Table 6:** Fossil samples used in symphyseal cross-sectional analysis.

*Australopithecus*	Pleistocene *Homo*
A.L.: 277–1, 198–1, 207–13, 288–1, 266–1, 333–6, 330–5, 400-1a, 315–22, 333w-12, 444–2, 437–1, 417-1a, 438-1g, 437–2, 620.1; MAK-VP-1/12; L.H. 44; KNM-KP 29281; KT12/h1	OH: 13, 22; Dmanisi: 211, 2600; KNM-ER: 730, 1802; KNM-WT 15000; Sangiran: 1, 5, 6; Zhoukoudian H-1; Ternifine (Tighinif): 1, 2, 3; Mauer; Krapina H; Arago: 2, 13; Montmaurin; Spy 1; La Chappelle aux Saints; Amud; La Ferrassie; Oberkassel: M, F; Predmost 3; Skhul 5; Zhoukoudian Upper Cave 104

### Stature estimates

The original stature estimates provided by Brown, Sutikna [1] were 1.04–1.09 m, with an average of 1.06 m based on African pygmies, based on analyses using least squares regression (LS), major axis (MA) and reduced major axis (RMA). These equations are from Jungers [113], who calculated stature in the small-bodied australopith (A.L. 288–1, “Lucy”), and the same pygmy training sample was used subsequently to assess competing extrapolations to short stature by Konigsberg, Hens [112]. Stature estimation for LB1 is revisited here in several different ways. We apply the classic calibration equation from a large-bodied modern human reference sample that was validated and recommended by Konigsberg et al. [112] using a femur length for LB1 of 280 mm. We also bracket stature estimates by application of both inverse (ordinary least squares regression of stature on femur length) and classic calibration (regression of femur length on stature, solving for stature) using the reference sample of 19 African pygmies discussed above; we repeat this for femur length plus tibia length (515 mm in LB1). At 216 mm, the tibia of a second adult tibia of *H*. *floresiensis* (LB8) is almost 2 cm shorter than that of LB1; if we use the crural index of LB1 (83.9) to estimate femur length for LB8, a value of just over 257 mm is obtained. As we did for LB1, we predict stature for LB8 from the human pygmy training sample using the sum of femur and tibia lengths (473 mm). The stature estimates for both LB1 and LB8 are compared with published data on stature in individuals with DS to address claims by Henneberg et al. [25] that stature in the Liang Bua fossils is expected for modern humans with this pathology.

These estimates are extrapolations because the lengths of LB1 tibia and particularly the femur are smaller than those recorded for small-bodied human populations from the Andaman Islands, Africa and the Philippines. More specifically, at 280 mm, the femur of LB1 is almost 4.7 standard deviations (sd) from the African pygmy mean (377 mm), 5.0 sd from the Negrito mean (378 mm), and 5.7 sd from the Andamanese mean (386 mm). Konigsberg, Hens [112] demonstrated that classic calibration works best in cases of when extrapolation is required to predict stature; this conclusion was reached empirically via validation on a pygmy test sample. The estimates from classic calibration are therefore our preferred estimates. No modern human population could serve as a reasonable prior given the very small size of the Liang Bua limb bones, including small humans from Asia, but using a small-bodied population is preferable as it requires the least amount of extrapolation (see also [137]).

The comparative data were drawn from growth charts for Turkish individuals with DS, chosen because they extended to age 18 and because they were among the shorter population for which data were available. Nevertheless, average statures for Turkish females (1.66 m) and males (1.76 m) were taller than local Javanese populations (1.50 m for females, 1.60 m for males; [138]). To generate rough estimates of the stature reduction associated with DS in the Javanese population, we applied an 11% (female) and 10% (male) decrease in height to the average euploid male and female statures based on the difference between the Turkish euploid and DS samples. These estimates should be viewed with caution, however, as the Turkish data were reported as centiles rather than population averages.

### Relative femoral length

We present summary data on the foot:femur ratio for LB1, as well as skeletal samples of modern humans of normal stature and short stature, from [7]. We are unaware of skeletons of adults diagnosed with Down syndrome, but analogous anthropometric data are available for adults with Down syndrome and for euploid controls in Smith and Ulrich [115], the raw data of which was provided by the corresponding author (E. Smith); the ratio of their “foot” to “thigh” lengths approximates the foot:femur index. We also present summary data in S3 Fig on the humerofemoral index based on adult osteological specimens for LB1, African Zulus, African pygmies, Andamanese, Asian Negritos and Khoe-San. Again, lacking skeletons of individuals with DS, we must turn to anthropometrics of living individuals for comparison; the ratio of mean upper arm length to mean thigh length can be calculated from Smith and Ulrich [115] for both individuals with DS and controls. These data permit us to evaluate claims that individuals with DS converge on LB1 in relative femoral length.

However, the mean soft tissue ratios of the euploid controls were consistently higher (58.7) than both the skeletal and fleshy foot:femur (not thigh) length ratios for normal humans in Jungers et al. [7], which were 53 (skeletal) and 54 (fleshy) regardless of body size. Soft tissue thigh length is apparently not precisely the same thing as skeletal femur length. To render the anthropometric and skeletal metrics commensurate, we therefore adjusted all soft tissue values downward by 5.6 points so that the euploid soft tissue mean ratio and mean skeletal ratios were equal at 0.53. A one-tailed t-test was used to evaluate the probability that the LB1 ratio was significantly higher than the DS sample average.

### Phalangeal length

Henneberg, Eckhardt [25] argued that manual phalanges, particularly the distal phalanges, of LB1 were short relative to European standards. They also cited the short absolute foot length in LB1 relative to modern humans as evidence of “short digits,” and therefore support for the DS diagnosis. Short fingers (brachydactyly) and short broad hands are frequently cited as part of the DS phenotype, but no quantitative measurements are available, and it is unclear whether this should be assessed in absolute or relative terms. Although we could not confirm that short toes is a clinical sign of DS, we nevertheless evaluated whether the LB1 phalanges (manual and pedal) were absolutely and relatively shorter than those of normal- and small-statured populations of euploid modern humans. Note that LB10 was originally described as a proximal pedal phalanx [32], but new fossils indicate that it is really a robust proximal pollical phalanx [139, 140]. Most proximal, intermediate and distal pedal and manual phalanges of LB1 and LB6 cannot be assigned to specific rays and are therefore compared to data from multiple digits from females of African and European ancestry in the Hamann-Todd (Cleveland Museum of Natural History) and Terry Collection (National Museum of Natural History) (n = ~98). A smaller and less complete data set of 22 individuals of mixed sex from small-bodied human populations from Asia (Musée de l’Homme, Paris) and Africa (University of Geneva) was also utilized. Summary statistics (mean, sd and range) are presented for both raw length measurements and relative length (scaled by femoral or humeral length as appropriate). As results are broadly comparable for the normal- and small-statured populations, only those of the latter are presented. All phalangeal and associated long bone data were provided by Dr. Campbell Rolian, University of Calgary.

## Supporting Information

S1 Fig Comparison of the LB1 endocast to DS and euploid virtual endocasts. (TIF)

S2 Fig Radiographs of LB6 mandible. (TIF)

S3 Fig Box plot of humerus: femur and upper arm: thigh ratios in euploid and DS samples and LB1. (TIF)

S1 Methods CT and MRI data of DS patients. (DOCX)

S1 Table Dental traits common in DS and their presence / absence in LB1 and LB6. (DOCX)

S2 Table Manual phalanx dimensions of average-sized modern humans and the Liang Bua hominins. (DOCX)

S1 Text Results of additional analyses. (DOCX)
