# Supplementary figures and images for: A Critical Evaluation of the Down Syndrome Diagnosis for LB1, Type Specimen of Homo floresiensis
Source: PLoS One. 2016 Jun 8;11(6):e0155731. doi: 10.1371/journal.pone.0155731 (PMC4898715; doi:10.1371/journal.pone.0155731)

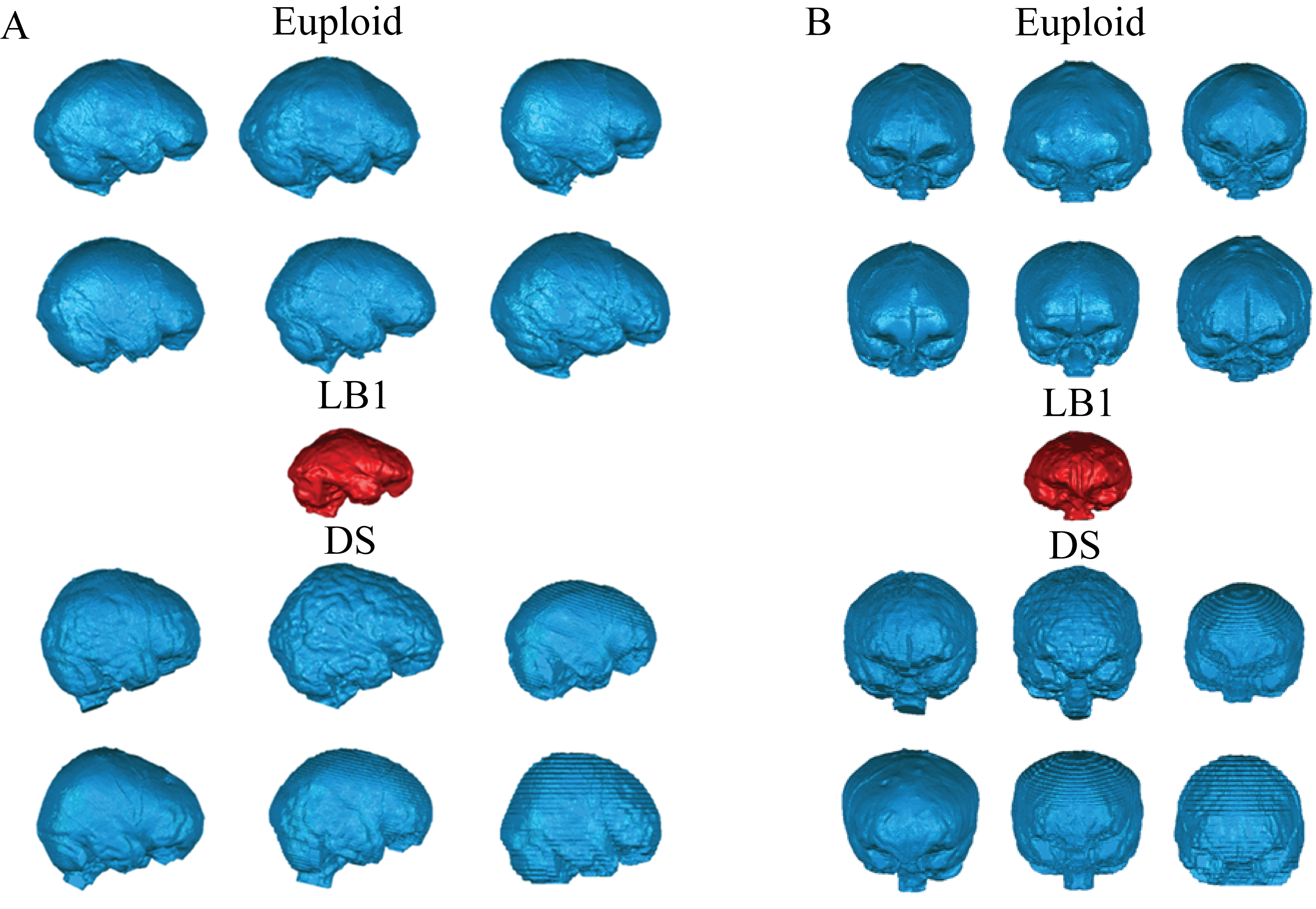

Supplement: S1 Fig — (TIF) [file pone.0155731.s001.tif]

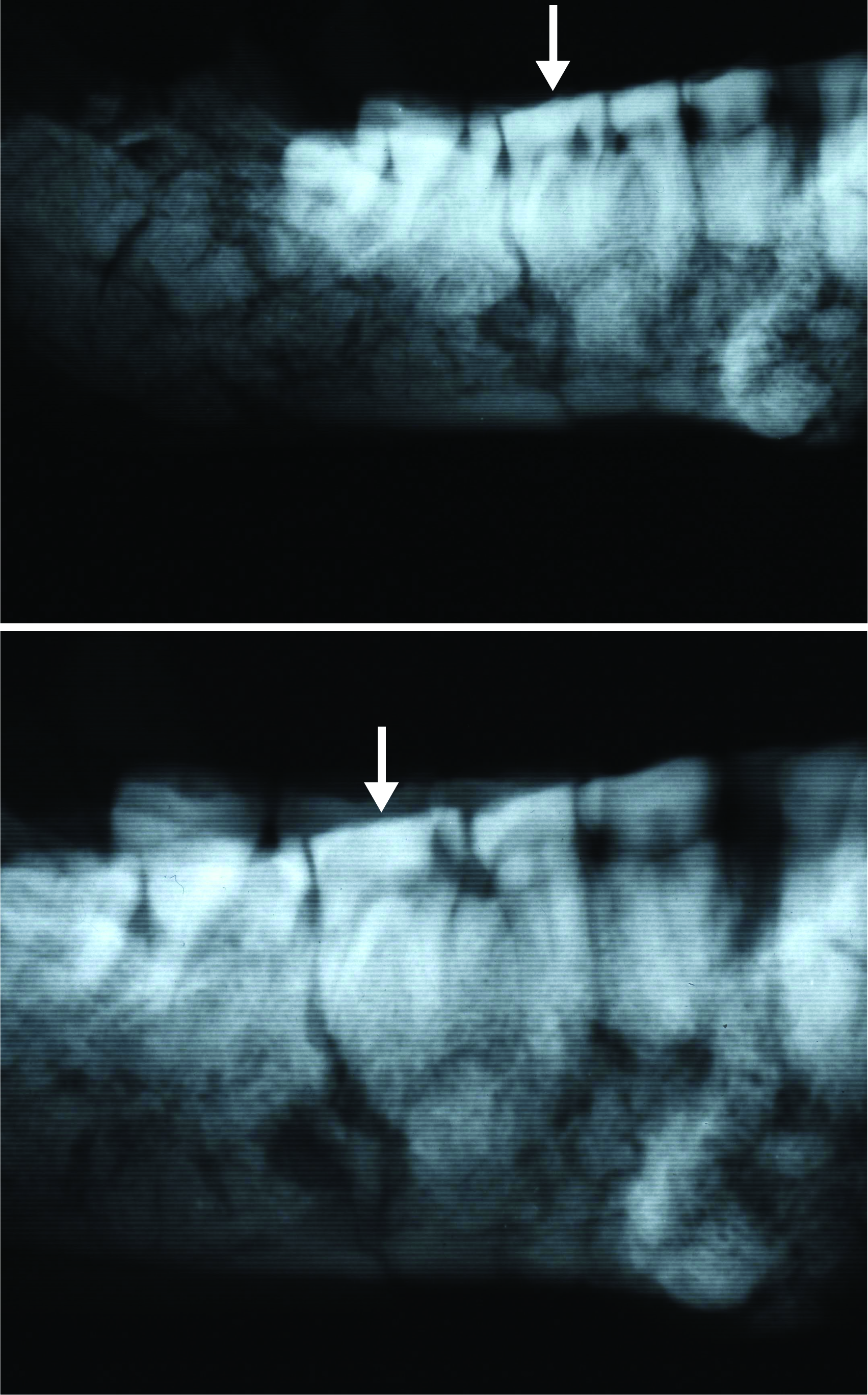

Supplement: S2 Fig — (TIF) [file pone.0155731.s002.tif]

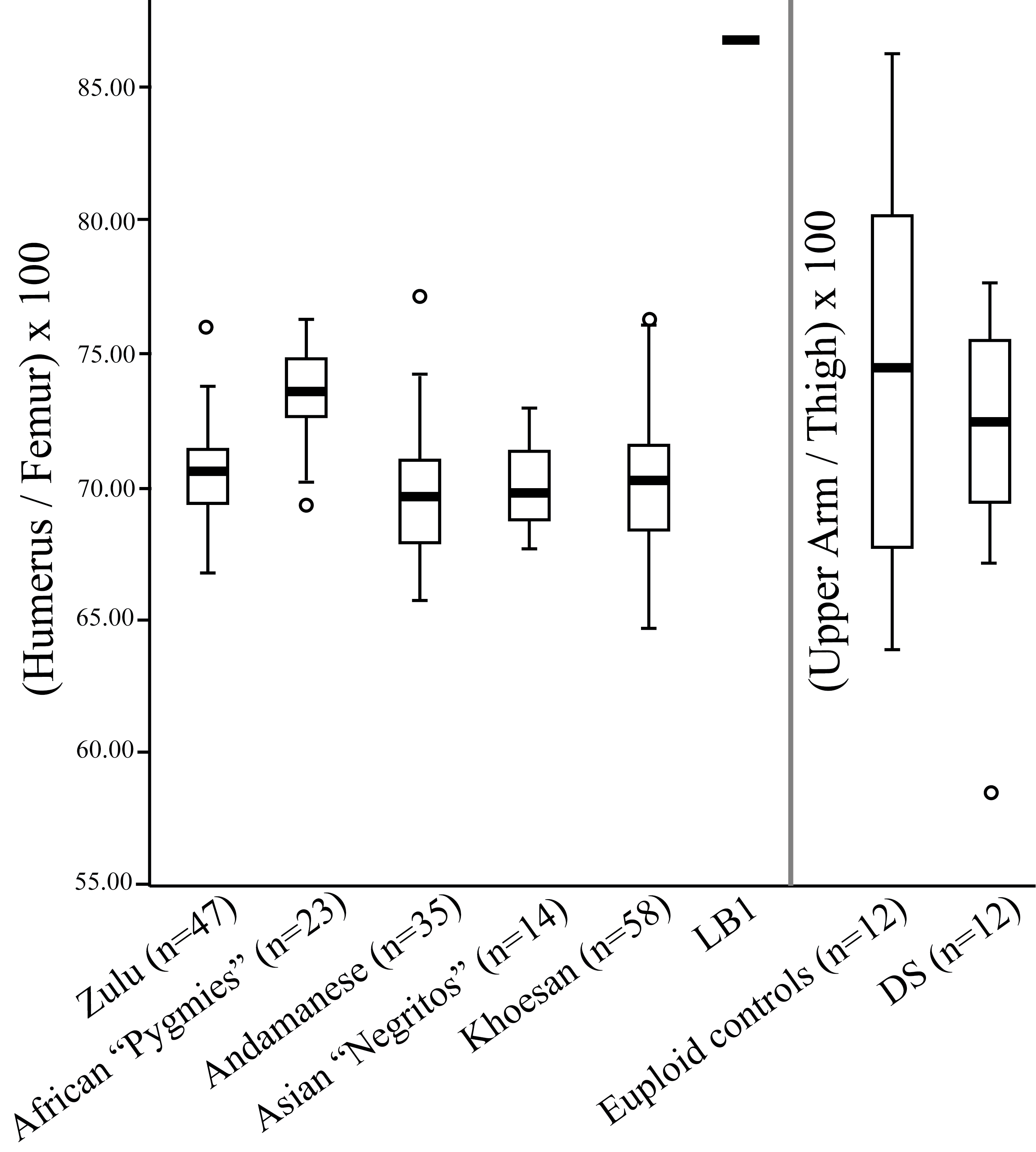

Supplement: S3 Fig — (TIF) [file pone.0155731.s003.tif]
