# Supplementary material for: A Critical Evaluation of the Down Syndrome Diagnosis for LB1, Type Specimen of Homo floresiensis
Source: PLoS One. 2016 Jun 8;11(6):e0155731. doi: 10.1371/journal.pone.0155731 (PMC4898715; doi:10.1371/journal.pone.0155731)
Supplement: S1 Methods — (DOCX) [file pone.0155731.s004.docx]

**S1 Methods**

*CT and MRI data of DS patients*

Following an internal review board (IRB) approved protocol, CT data were mined from the clinical repository at Barnes-Jewish-Christian Hospital, in St. Louis, Missouri. Acceptance criteria required the subject to have a clinical diagnosis of DS, a full head CT scan with a resolution of 3 mm or less in the cranial-caudal direction, and have a radiology report that indicated no abnormal morphology for a person with DS. Selected cases were de-identified using the Radiological Society of North America’s Clinical Trials Processing software and transferred to a secure data repository for subsequent analysis. We also acquired MRI data from 21 adult individuals with DS from Richard Haier’s research group at University of California, Irvine. MRI data were processed as follows: 1) gray scale values were inverted to highlight bone, cerebrospinal fluid and air, 2) a threshold was determined and set to represent the outer skull surface, 3) the background was isolated using mathematical morphology and region growing operations, and 4) the image data were manually edited to refine the skull segmentation if needed, and 5) a connect and keep operation was used to define the external skull surface. Once the skull was fully segmented within the image data, a 3D object was created using the “optimal” setting within the Calculate 3D module of Mimics. This model was exported as a triangulated model in .ply format. MRI data are not ideal for visualizing bone, and surface renderings from the MRI data are generally of low quality. Therefore, although we processed data for 28 individuals with DS in total, not all were used in the analyses due to segmentation issues.
