# Supplementary material for: A Critical Evaluation of the Down Syndrome Diagnosis for LB1, Type Specimen of Homo floresiensis
Source: PLoS One. 2016 Jun 8;11(6):e0155731. doi: 10.1371/journal.pone.0155731 (PMC4898715; doi:10.1371/journal.pone.0155731)
Supplement: S1 Table — (DOCX) [file pone.0155731.s005.docx]

**S1 Table. Dental traits common in DS and their presence / absence in LB1 and LB6.**

| Trait | DS | LB1 | LB6 mandible |
| --- | --- | --- | --- |
| microdontia | 35-55%^1^ | No^2^ | No^3^ |
| peg-shaped I^2^/canines | 10-25%^4^ | No^2^ | No*^3^ |
| oligodontia | >60%^5^ | No^2^ | No*^3^ |
| tooth transposition (C-P^1^) | 15%^6^ | No^2^ | No*^3^ |
| delayed development |  | No^2^ | No^3^ |
| retained primary teeth | 15-34%^7^ | No^2^ | No^3^ |
| agenesis M3 | >70%^8^ | R M^3 2^ | No*^3^ |
| agenesis excluding M3 (hypodontia) | >50%^9^ | R P_4_? ^2^ | No*^3^ |
| impaction (maxillary C) | 15%^10^ | No^2^ | No*^3^ |
| relatively small palate | Yes^12^ | No^2^ | No*^3^ |
| Angle’s class III malocclusion | >60%^13^ | No^2^ | No*^3^ |
| anterior open bite | Yes ^14^ | No^2^ | No*^3^ |
| maxillary incisor protrusion | >70%^15^ | No^4,5^ | No*^5,6^ |
| mandibular incisor protrusion | >80%^16^ | No^4,5^ | No*^5,6^ |
| bimaxillary dental protrusion | Yes ^17^ | No^4,5^ | No*^5,6^ |
| fluctuating dental asymmetry | Yes ^18^ | No^4,5^ | No^5,6^ |
| hypocalcification | Yes ^19^ | No^4,5^ | No^5,6^ |
| thin enamel permanent dentition | Yes ^20^ | No^4,5^ | No^5,6^ |
| taurodontism | >35%^21^ | ? | ? |
| bruxism | >40%^22^ | ? | ? |

*Not supported by the arch dimensions, occlusion and tooth wear in the LB6 mandible [19].

1 [49-51] 2 [19,52]; 3 [19,53]; 4 [54,55]; 5 [56-61]; 6 [54,62]; 7 [63,64]; 8 [54,65]; 9 [54,66]; 10 [54,66]; 11 [67,68]; 12 [69,70]; 13 [49,67]; 14 [67,71,72]; 15 [73]; 16 [72,73]; 17 [72]; 18 [74]; 19 [75]; 20 [50]; 21 [41,76]; 22 [77,78]
