# Supplementary material for: A Critical Evaluation of the Down Syndrome Diagnosis for LB1, Type Specimen of Homo floresiensis
Source: PLoS One. 2016 Jun 8;11(6):e0155731. doi: 10.1371/journal.pone.0155731 (PMC4898715; doi:10.1371/journal.pone.0155731)
Supplement: S2 Table — (DOCX) [file pone.0155731.s006.docx]

**S2 Table. Absolute and relative length of manual phalanges in the Liang Bua hominins and a comparative sample of average-sized modern humans.**

| Manual Phalangeal Length | | | | |
| --- | --- | --- | --- | --- |
| Modern Humans | | | | Liang Bua values |
| Digit | Average | SD | Range |  |
| Proximal | | | | Indeterminate digit - LB1:>33.4; LB6: 31.2 |
| II | 39 | 2.6 | 32.9-45.0 | Digit I - LB10: 24.2 |
| III | 43.7 | 3 | 37.5-51.2 |  |
| IV | 40.9 | 2.8 | 34.7-47.6 |  |
| V | 32.1 | 2.3 | 26.9-37.9 |  |
| Intermediate | | | | Indeterminate digit - LB1: 25.6; LB6: 16.9 |
| II | 23.1 | 1.7 | 19.2-27.4 |  |
| III | 28.3 | 2.2 | 22.7-33.7 |  |
| IV | 27.1 | 2.1 | 21.7-32.2 |  |
| V | 19.6 | 2.1 | 14.8-27.3 |  |
| Distal | | | | Indeterminate digit - LB1: 13.4; LB6: (?)10.5-12.9 |
| I | 22.6 | 2 | 18.5-26.9 | Digit I - LB1: 15.2 |
| III | 18.7 | 1.5 | 15.4-22.4 |  |
| V | 16.5 | 1.3 | 13.1-19.5 |  |
|  |  |  |  |  |
| Manual Phalangeal Length as % of Humeral Length (LB1 only) | | | | |
| Modern Humans | | | | Liang Bua values |
| Digit | Average | SD | Range |  |
| Proximal | | | | Indeterminate digit - >13.7% |
| II | 12.8% | 0.7% | 11.3-14.5% |  |
| III | 14.4% | 0.9% | 12.4-16.5% |  |
| IV | 13.4% | 0.8% | 11.5-15.2% |  |
| V | 10.6% | 0.6% | 9.1-11.8% |  |
| Intermediate | | | | Indeterminate digit - 10.5% |
| II | 7.6% | 0.5% | 6.6-9.4% |  |
| III | 9.3% | 0.6% | 8.1-11.0% |  |
| IV | 8.9% | 0.6% | 7.6-10.2% |  |
| V | 6.4% | 0.6% | 5.2-8.4% |  |
| Distal | | | | Indeterminate digit - 5.5% |
| I | 7.4% | 0.6% | 6.0-8.8% | Digit I - 6.3% |
| III | 6.2% | 0.5% | 5.3-7.3% |  |
| V | 5.4% | 0.4% | 4.6-6.4% |  |
