# Supplementary material for: A Critical Evaluation of the Down Syndrome Diagnosis for LB1, Type Specimen of Homo floresiensis
Source: PLoS One. 2016 Jun 8;11(6):e0155731. doi: 10.1371/journal.pone.0155731 (PMC4898715; doi:10.1371/journal.pone.0155731)
Supplement: S1 Text — (DOCX) [file pone.0155731.s007.docx]

**S1 Text**

**Results of Additional Analyses**

*Cranium and Brain*

Head circumference: Falk et al. [1] first published a measurement of head circumference (HC; a proxy for brain size) of 39.4 cm. Henneberg et al. [2] provided a similar measurement of 38.5 cm and then estimated HC in the local Rampasasa population from its average endocranial volume (EV) by a linear regression equation developed from adult Aboriginal Australians. From the 95% confidence interval of 450-522 mm, and a SD of 20.5 mm based on the Australian sample, they determined LB1 HC to be -3.17 to -6.68 SDs below the Rampasasa range. They argued that this range was comparable to the deviation of HCs in a sample of DS children (15 mo to 13.9 y) from same-aged population averages found in Castells et al. [3]. We find this approach unnecessarily convoluted. Head circumference data are available for adult individuals with DS. For example, Tüysüz et al. [4] presented a growth curve for Turkish females with DS from ages 3-18 years. At age 18, the 97^th^, 50^th^ and 3^rd^ percentiles are 54.2, 51.3 and 48.6 cm, respectively. By comparison, the LB1 soft tissue values (estimated from the osteological measurements using the formula provided by Henneberg et al. [2014]) are 42.2 based on Falk et al. [1] and 41.3 cm based on Henneberg et al. [2], both well below even the 3^rd^ percentile of estimated HC for DS. Comparable data were not available for Indonesian populations. In addition to the much smaller brain size of LB1, the shape of the LB1 endocast is distinct when compared visually with the virtual endocasts from the six DS females in our sample and six non-DS females (S1 Fig).

**S1 Fig. Right-lateral (A) and anterior (B) views of virtual endocasts for six Euploid females from a previous study [5], LB1, and our six subjects with DS.** All virtual endocasts are scaled to size.

Cranial asymmetry in Down syndrome: It has been hypothesized that trisomy 21 causes "amplified developmental instability" and increased variance of phenotypic characteristics [6-8]. Developmental instability (DI) refers to an organism's tendency to produce a morphological change in response to developmental perturbations. Proponents of the hypothesis that Down syndrome is a case of amplified developmental instability argue that trisomy 21 causes a generalized genetic imbalance that disrupts evolutionarily conserved developmental pathways by decreasing developmental homeostasis and precision.

Historically, DI has been estimated as a measure of the metric differences between the left and right sides of symmetrically developing organisms (i.e., fluctuating asymmetry). Since in typically developing symmetric organisms, the left and right halves experience the same environmental and genomic influences during development, bilateral features are expected to develop as mirror images of the opposite side. According to this model, the developmental program of any organism is driven genetically and buffered or canalized in such a way that development tends to proceed along evolved trajectories unless perturbed by environmental or genetic insult. Genetic or environmental stress during development can increase developmental noise, expressed as minor departures from an ideal developmental program of perfect bilateral symmetry. While symmetry is a property of the individual, patterns of asymmetry for specific traits are studied at the level of the population [9-12].

Starbuck et al. [13] tested a hypothesis of developmental instability in DS by estimating fluctuating asymmetry in the 3D morphology of soft tissue facial features in children with DS and found that parts of the face in individuals with DS, especially those derived from the mandibular prominence of the first pharyngeal arch, showed increased developmental instability as measured by fluctuating asymmetry relative to carefully chosen comparative (euploid) samples. Henneberg et al. [2] used the correspondence between their facial measures of asymmetry in the LB1 specimen and the soft tissue facial features evaluated by Starbuck et al [13] as support for the diagnosis of DS. However, these two sets of measurements are not commensurate. The data from LB1 were measures of directional asymmetry in osteological features from a single fossil [14], not measures of fluctuating asymmetry in soft tissue features measured across a sample.

*Dentition*

The clinical literature regarding oral health issues (related to both hard and soft tissues) is extensive. While occlusal issues related to the underdevelopment of the maxillae are common, the frequency and expression of other traits is highly variable and no traits are DS specific. Although, they ignore the larger set of dental traits more often associated with DS [15] (S1 Table), with evidence preserved in the teeth and dental arches of LB1 and LB6, Henneberg et al. ([2]: SI) argue that the presence of periodontitis, a low caries incidence and possible evidence of taurodontism in LB1 *H. floresiensis* is consistent with DS.

Periodontal disease: A higher frequency of periodontal disease has been documented for DS patients than in age-matched controls, with prevalence rates as high a 58% of individuals 9-39 years being reported, versus ~20% of the control group [16]. The etiology of this is uncertain, but factors of poor oral hygiene, immunological deficiency, impaired masticatory function and salivary deficiency have been proposed [17,18]. Evidence of periodontal disease in LB1 includes heavy dental calculus and alveolar recession in the posterior dental arch of the mandible and maxilla [19]. There is no evidence of periodontal disease in the LB6 mandible.

Periodontal disease in LB1 has previously been discussed in Brown and Maeda [19], with calculus deposits and alveolar bone erosion associated with the posterior teeth. The formation of static plaque deposits and calculus is multifactorial, and periodontal disease is common in pre-Neolithic and post-Neolithic human populations [20,21], some species of wild primates [22,23], and has been recorded in hominins as diverse as South African australopiths [24] and *Homo* *antecessor* [25]. In wild, non-carnivore, mammals, the frequency of periodontal disease is linked to diet [26], with heavy tartar deposits in foliverous colobines [23]. While the presence of infant Stegodon in the Pleistocene deposits associated with *H. floresiensis* at Liang Bua, suggests the seasonal consumption of some meat, there is no direct evidence of the vegetative component of the diet [19]. The reasons for the adherence of calculus deposits in LB1 remain unknown, but are likely to involve both oral physiology and diet. By itself, and in the absence of numerous other dento-facial indicators (S1 Table), the presence of periodontal disease in LB1, but not LB6, adds very little to a differential diagnosis of DS.

Caries: Although they have previously indicated that they believe that tooth loss in LB1 (LB1 right P2, right M3) was the result of caries [27], Henneberg et al. ([2]: Table 1, SI) argue that a low caries incidence in LB1 and LB6 is consistent with DS. In general, DS children appear to have a lower caries incidence than reported for their unaffected siblings [28]. Although minimal tooth wear has usually preserved the fissures and crevices on their occlusal surfaces, it seems that the oral environment in DS children is not conducive to the adherence of *S. mutans* biofilms and fermentation of caries producing lactic acid [18]. This contrasts with a low caries incidence in recent hunter-gatherers, and Pleistocene hominins, that is associated with heavy tooth wear and diets low in fermentable carbohydrates [29,30,31]. There is no evidence of caries in LB1, or the LB6 mandible, and the pattern of tooth wear, on both the occlusal and interproximal surfaces, is consistent with that in hunter-gatherers and Pleistocene hominins [19,32,33].

Taurodontism: In 2008 Obendorf et al. [34] argued that the Liang Bua *H. floresiensis* skeletons were most likely the remains of modern humans with myxoedematous endemic (ME) cretinism. They also claimed to see evidence of taurodontism in the LB1 and LB6 teeth in “captured images from X-ray scans presented in The mystery of the human hobbit (BBC Horizon, 2005)” ([35], p. 1290; [36]). Some of these images can be seen in S2 Fig.

Taurodont molar teeth have a vertically enlarged pulp chamber, with apical displacement of the pulpal floor, no constriction at the level of the cement enamel junction, the point of trifurcation/bifurcation of the roots is moved apically, and there is greatly reduced vertical root length Third molar teeth are more commonly affected than the first and second molars, both deciduous and permanent teeth can have taurodont pulp chambers [37] and permanent premolars can also be taurodont [38]. Taurodont teeth are known to occur at high frequency in a range of x-linked syndromes [39,40], DS [41,42], Klinefelter’s Syndrome [43], and at a lower frequency in the human population more broadly, although some modern human populations have higher frequencies of taurodontism than others [44,45]. For instance, a high frequency of taurodontism has been described for at least one Australomelanesian population, the Broadbeach burials [45] [contra 2]. Taurodont permanent and deciduous teeth are also known to occur at a high frequency in Neanderthals [46], for instance at Krapina [47], and in Zhoukoudian *H. erectus* [48], without any associated skeletal evidence of developmental syndromes.

Henneberg et al. ([2], Table 1, SI) revisit the subject of taurodontism in the Liang Bua hominins, using this as possible support for their DS diagnosis. They write, “Others (Obendorf et al. 2008) have proposed the presence in LB1 of **“**significant taurodontism; that is, the presence of an enlarged tooth body due to an apical displacement of the root bifurcation, is evident in these and other LB1 teeth. … we regard the presence of some degree of taurodontism in LB1 as sustainable. Its presence is, however, uncertain due to the poor quality of evidence that continues to be provided by Peter Brown and his supporters.”

However, micro CT scans of the LB1 mandibular first molars demonstrate that they are not taurodont [33,36]. Similarly, radiographs of the LB6 mandible demonstrate that the M_1_’s have normal pulp and root dimensions, but shadows from antimeres obscure these details in the other molar teeth (S2 Fig).

**S2 Fig. Lateral radiographs of LB6 posterior teeth and tooth roots.** Both images show that the M_1_ (indicated by the arrow) is not taurodont.

*Postcranial anatomy*

Humerus:Femur ratio: Henneberg et al. [2] asserted that the femur was short relative to the humerus in LB1 and individuals with DS. They based this on data on growth in Indian children with DS (6-18 y) and anthropometric measurements of the thigh and upper arm from Smith and Ulrich [79] on matched adult DS and euploid samples. However, the average ratio was actually lower in DS than the matched controls in the adult dataset from Smith and Ulrich [79], which indicated that, if anything, the arm was disproportionately short rather than the leg in DS (SI Fig 3). Moreover, the ranges for the anthropometric (soft tissue) ratios (raw data from Ref. [79] provided by E. Smith) are inflated relative to the skeletal measures and likely incorporate a high degree of measurement error. In fact, the range of soft tissue values for the control (euploid) sample subsumes the equivalent measures for the DS sample as well as the skeletal ranges for 1 average-sized and 4 small-bodied modern humans groups. The LB1 value of 86.8 is considerably higher than the average values for both the skeletal and anthropometric ratios reported for the euploid and DS groups and well beyond the range documented for DS. Together, these observations suggest that 1) the available data for upper arm:thigh ratio do not discriminate between the DS and the control group and 2) LB1 is likely an outlier with respect to both groups, but skeletal ratios for DS would be necessary to confirm this.

**S3 Fig. Humerus: femur (skeletal) and upper arm: thigh (anthropometric) ratios of euploid and DS samples as well as LB1.** The five samples on the left represent one average-sized (Zulu) and four small-bodied populations of euploid humans. The ratios for the DS and control samples on the right were based on soft-tissue measurements from a clinical study [79]. The box-and-whisker plots include the average, +/- 1 standard deviation and range (extreme outliers indicated by circles).

Digit length: Additional comparative data on absolute and relative phalangeal length from a cadaver sample of average body size are provided in S2 Table to complement the smaller dataset derived from small-bodied populations in Table 3 of the main text.

Atlantoaxial or atlanto-occipital instability. Atlantoaxial and atlanto-occipital (or craniocervical) instability and hypermobility have been reported for DS, but are difficult to assess reliably radiologically [80]. Both are usually attributed to ligamentous laxity (which is pervasive in patients with DS) (e.g., [81]) as much of the stability of these joints is through soft tissue mechanisms. Bony abnormalities sometimes associated with atlantoaxial instability are hypoplasia of the C1 arch, basilar invagination and os odontoideum [80]. Flattening or hypoplasia of the occipital condyles can create a “rocker bottom” which also contributes to atlanto-occipital instability [82,83].

Henneberg et al. [2] described LB1 as having an “atlanto-occipital abnormality” and cited the work of Kaifu et al. [84] who described the right occipital condyle of LB1 as slightly concave and bearing a depression on its posterior portion, along with a roughened articular surface of the corresponding superior articular facet of C1. Kaifu et al. [84] interpreted this irregular anatomy as indicative of ***limited*** mobility (rather than instability and hypermobility), possibly from asymmetrical insertion of the nuchal muscles or congenital torticollis. Regardless of its interpretation, this description of the occipital condyle does not correspond to the “rocker bottom” anatomy sometimes seen in atlanto-occipital instability, although we could not locate any photographs with which to compare the LB1 condition. Therefore, while there is an “abnormality,” there is no evidence that this corresponds to the specific anatomical anomaly seen in some individuals with DS.

Feet: The “flat foot” feature identified by Henneberg et al. [2] in LB1 and in DS is comparable in name only. Flat foot (pes planus or pes valgus) is found in 60-70% of pediatric DS patients aged 4-10 years [85], which is higher than values reported for similarly aged non-DS children (24-40%) [86,87], and adults with DS (20%) [88]. Most cases of flat foot in DS are categorized as flexible flat foot, which refers to the loss of the medial (longitudinal) arch of the foot during weight support, often accompanied by pronation of the foot. This is again likely related to ligamentous laxity in DS [89]. Rarely flat foot in DS results from vertical talus (0.7%; [88]) which is characterized by a dislocation of the navicular dorsally on the talus, a talus that is waisted (hour-glass shaped), a calcaneus that narrows anteriorly and is beak-shaped, and a navicular that is wedge-shaped on its plantar side [90,91]. Other common foot anomalies in DS include metatarsus primus varus (40% of children) and hallux valgus (26%), or both (34%) [85] which may be related to the common clinical finding of a wide gap between the first two toes (“sandal gap”).

The talus and navicular (but not the calcaneus) are preserved for LB1 and do not show evidence of vertical talus [92,93]. The flexible flat foot seen in DS could not be assessed in LB1 as this is not a bony deformity, but rather relates to ligamentous laxity. LB1 has been described as having a “flat foot” in the sense of a phylogenetically primitive architecture that is also seen in apes, some australopiths and possibly early *Homo* [92,93]. This interpretation hinged primarily on the large medial navicular tuberosity and lateral wedging of the navicular in LB1 which is generally interpreted as weight-bearing in apes and other fossil hominins that exhibit this feature [94]. There was no discussion of a large navicular tuberosity associated with DS in the clinical literature.

Metatarsus primus varus is identified when the intermetatarsal angle between the first two metatarsals is >9⁰ [95]. Alignment of the articular surfaces of the medial cuneiform and first metatarsal (first tarso-metatarsal joint) indicate that the first metatarsal is fully adducted and in line with the other metatarsals [92] in contrast with the expected abduction that defines metatarsus primus varus. Hallux valgus is identified when the hallux valgus angle exceeds 15⁰ [95]. This could not be evaluated for LB1 as the 1^st^ proximal phalanx was not preserved. However, these two conditions are associated and often co-occur [95,96].

The presence of osteophytes in two proximal pedal phalanges was used as supporting evidence for flat foot in LB1 [2]. It is difficult to know the etiology of these osteophytes as they are a common feature of osteoarthritis which is influenced by many factors [97]. It may be the case that the flat foot of LB1 impacted their development. This is not, however, support for a DS diagnosis because the phylogenetically primitive traits leading to flat foot in this individual are very distinct from the flexible flat foot found in DS. Osteophytes could also be a result of trauma.

1. Falk D, Hildebolt C, Smith K, Jungers WL, Larson SG, et al. (2009) The type specimen (LB1) of *Homo floresiensis* did not have Laron syndrome. Am J Phys Anthrop 140: 52-63.

2. Henneberg M, Eckhardt RB, Chavanaves S, Hsü KJ (2014) Evolved developmental homeostasis disturbed in LB1 from Flores, Indonesia, denotes Down syndrome and not diagnostic traits of the invalid species *Homo floresiensis*. Proc Natl Acad Sci USA 111: 11967-11972.

3. Castells S, Torrado C, Bastian W, Wisniewski KE (1992) Growth hormone deficiency in Down's syndrome children. J Intellectual Disabil Res 36: 29-43.

4. Tüysüz B, Göknar NT, Öztürk B (2012) Growth charts of Turkish children with Down syndrome. Am J Med Genet A 158A: 2656-2664.

5. Falk D, Hildebolt C, Smith K, Morwood MJ, Sutikna T, et al. (2007) Brain shape in human microcephalics and *Homo floresiensis*. Proc Natl Acad Sci USA 7: 2513-2518.

6. Shapiro BL (1970) Prenatal dental anomalies in Mongolism: Comments on the basis and implications of variability. Ann NY Acad Sci 171: 562-577.

7. Shapiro BL (1975) Amplified developmental instability in Down's syndrome. Ann Hum Genet 38: 429-437.

8. Shapiro BL, Hermann J, Opitz JM (1983) Down syndrome—a disruption of homeostasis. Am J Med Genet A 14: 241-269.

9. Palmer AR, Strobeck C (1992) Fluctuating asymmetry as a measure of developmental stability: Implications of non-normal distributions and power of statistical tests. Acta Zool Fennica 191: 57-72.

10. Palmer AR, Strobeck C (2003) Fluctuating asymmetry analyses revisited. In: Polak M, editor. Developmental Instability (DI): Causes and Consequences. New York: Oxford University Press. pp. 279-319

11. Palmer AR, Strobeck C (1986) Fluctuating Asymmetry: Measurement, Analysis, Patterns. Ann Rev Ecol Syst 17: 391-421.

12. Palmer AR (1994) Fluctuating asymmetry analysis: A primer. In: Markow T, editor. Developmental Instability: Its Origins and Evolutionary Implications. Dordrecth, Netherlands: Kluwer.

13. Starbuck JM, Cole TM, Reeves RH, Richtsmeier JT (2013) Trisomy 21 and facial developmental instability. Am J Phys Anthrop 151: 49-57.

14. Jacob T, Indriati E, Soejono RP, Hsü K, Frayer DW, et al. (2006) Pygmoid Australomelanesian *Homo sapiens* skeletal remains from Liang Bua, Flores: population affinities and pathological abnormalities. Proc Natl Acad Sci USA 103: 13421-13426

15. Cheng RHW, Leung WK, Corbet EF, King NM (2007) Oral health status of adults with Down syndrome in Hong Kong. Spec Care Dentist 27: 134-138.

16. Saxén L, Aula S, Westermarak T (1977) Periodontal disease associated with Down’s syndrome: an orthopantomographic evaluation. J Periodontol 48: 337-341.

17. Amano A, Murakami J, Akiyama S, Morisaki I (2008) Etiologic factors of early-onset periodontal disease in Down syndrome. Jpn Dent Sci Rev 44: 118-127.

18. Madigan MT, Mantinko J, Bender KS, Buckley DH, Stahl DA (2015) Brock biology of microorganisms. Boston: Pearson.

19. Brown P, Maeda T (2009) Liang Bua *Homo floresiensis* mandibles and mandibular teeth: a contribution to the comparative morphology of a new hominin species. J Hum Evol 57: 571-596.

20. Arensberg B (1996) Ancient dental calculus and diet. Human Evolution 11: 139-145.

21. Eshed V, Gopher A, Hershkovitz I (2006) Tooth wear and dental pathology at the advent of agriculture: New evidence from the Levant. Am J Phys Anthrop 130: 145-159.

22. Kilgore L (1989) Dental pathology in then free ranging chimpanzees from Gombe National Park, Tanzania. Am J Phys Anthrop 80: 219-227.

23. Crovella S, Ardito G (1994) Frequencies of oral pathologies in a sample of 767 non-human primates. Primates 35: 225-230.

24. Ripamonti U (1989) The hard evidence of alveolar bone loss in early hominids of Southern Africa. J Periodontol 60: 118-120.

25. Lopez-Valverde A, Lopez-Cristia M, Gomez de Diego R (2012) Europe's oldest jaw: evidence of oral pathology. Br Dent J 212: 243-245.

26. Sone K, Koyasu K, Tanaka S, Oda S (2005) Effects of diet on the incidence of dental pathology in free living caviomorph rodents. Arch Oral Biol 50: 323-331.

27. Henneberg M, Eckhardt RB, Scholfield J (2010) The Hobbit trap: how new species are invented. Walnut Creek, CA: Left Coast Press.

28. Orner G Dental caries experience among children with Down's syndrome and their sibs. Arch Oral Biol 20: 627-634.

29. Campbell TD (1925) Dentition and palate of the Australian Aboriginal. Adelaide: Hassell Press.

30. Molnar S, McKee JK, Molnar I (1983) Measurements of Tooth Wear Among Australian Aborigines: I. Serial Loss of the Enamel Crown. American Journal of Physical Anthropology 61: 51-65.

31. Murphy TR (1968) The progressive reduction of tooth cusps as it occurs in natural attrition. Dental Practitioner 19: 483-488.

32. Brown P, Sutikna T, Morwood MJ, Soejono RP, Jatmiko, et al. (2004) A new small-bodied hominin from the Late Pleistocene of Flores, Indonesia. Nature 431: 1055-1061.

33. Jungers WL, Kaifu Y (2011) On dental wear, dental work, and oral health in the type specimen (LB1) of Homo floresiensis. Am J Phys Anthrop 145: 282-289.

34. Obendorf PJ, Oxnard CE, Kefford BJ (2008) Are the small human-like fossils found on Flores human endemic cretins? P R Soc B 275: 1287-1296.

35. Obendorf PJ, Oxnard CE, Kefford BJ (2008) Are the small human-like fossils found on Flores human endemic cretins? Proc R Soc B 275: 1287-1296.

36. Brown P (2015) Taurodont teeth in Homo floresiensis. http://peterbrown-palaeoanthropology.net.

37. Rao A, Arathi R (2006) Taurodontism of deciduous and permanent molars. J Indian Soc Pedod Prev Dent 24: 42-44.

38. Llamas R, Jimenez-Planas A (1993) Taurodontism in premolars. Oral Surg Oral Med Oral Pathol 75: 501-505.

39. Gage JP (1978) Taurodontism and enamel hypomaturation associated with X-linked abnormalities. Clinical Genetics 14: 159-164.

40. Jaspers MT, Witkop CJ (1980) Taurodontism, an isolated trait associated with sydndromes and x-chromosomal aneuploidy. Am J Hum Genet 32: 396-413.

41. Bell J, Civil CR, Townsend GC, Brown RH (1989) The prevalence of taurodontism in Down's syndrome. J Intellectual Disabil Res 33: 467-476.

42. Jaspers MT (1981) Taurodontism in the Down syndrome. Oral Surg Oral Med Oral Pathol 51: 632-636.

43. Gardner DG, Girgis SS (1978) Taurodontism, shovel-shaped incisors and the Klinefelter syndrome. Dental Journal 44: 372-373.

44. Constant DA, Grine FE (2001) A review of taurodontism with new data on indigenous southern African populations. Arch Oral Biol 46: 1021-1029.

45. Elvery MW, Savage NW, Wood WB (1998) Radiographic Study of the Broadbeach Aboriginal Dentition. Am J Phys Anthropol 107: 211-219.

46. Benazzi S, Nguyen HN, Kullmer O, Hublin J-J (2015) Exploring the biomechanics of taurodontism. J Anat 226: 180-188.

47. Kallay J (2014) A radiographic study of the Neanderthal teeth from Krapina. In: Brothwell DR, editor. Dental Anthropology: Volume V: Society for the study of human biology: Elsevier. pp. 75-86.

48. Xing S (2012) Morphological variation of Zhoukoudian Homo erectus teeth [PhD]: Graduate School, Chinese Academy of Science.

49. Oliveira ACB, Paiva SM, Campos MR, Czeresnia D (2008) Factors associated with malocclusions in children and adolescents with Down syndrome. Am J Orthod Dentofac Orthop 133: 489.e481-489.e488.

50. Bell E, Townsend G, Wilson D, Kieser J, Hughes T (2001) Effect of Down syndrome on the dimensions of dental crowns and tissues. Am J Hum Biol 13: 690-698.

51. Townsend GC (1983) Tooth size in children and young adults with trisomy 21 (Down) syndrome. Arch Oral Biol 28: 159-166.

52. Brown P, Sutikna T, Morwood MJ, Soejono RP, Jatmiko, et al. (2004) A new small-bodied hominin from the Late Pleistocene of Flores, Indonesia. Nature 431: 1055-1061.

53. Morwood MJ, Brown P, Jatmiko, Sutikna T, Wahyu Saptomo E, et al. (2005) Further evidence for small-bodied hominins from the Late Pleistocene of Flores, Indonesia. Nature 437: 1012-1017.

54. Shapira J, Chaushu S, Becker A (2000) Prevalence of tooth transposition, third molar agenesis, and maxillary canine impaction in individuals with Down syndrome. Angle Orthod 70: 290-296.

55. Gorlin RJ, Cohen MM, Hennekam RCM (2001) Syndromes of the head and neck: Oxford University Press.

56. Aksakalli S, Ileri Z (2012) Management of dental-orthopedic problems in down syndrome. European Journal of General Dentistry 1: 58-62.

57. Andersson E-MM, Axelsson S, Austeng ME, Øverland B, Valen IE, et al. (2014) Bilateral hypodontia is more common than unilateral hypodontia in children with Down syndrome: a prospective population-based study. The European Journal of Orthodontics 36: 414-418.

58. Kim JW, Sirnmer JP, Lin BPJ, Hu JCC (2006) Novel MSX1 frameshift causes autosomal-dominant oligodontia. J Dent Res 85: 267-271.

59. Kumasaka S, Miyagi A, Sakai N, Shindo J, Kashima I (1997) Oligodontia: A radiographic comparison of subjects with Down syndrome and normal subjects. Spec Care Dentist 17: 137-141.

60. Reuland-Bosma W, Reuland MC, Bronkhorst E, Phoa KH (2010) Patterns of tooth agenesis in patients with Down syndrome in relation to hypothyroidism and congenital heart disease: An aid for treatment planning. Am J Orthod Dentofac Orthop 137: 584.e581-584.e589.

61. Acerbi AG, de Freitas C, de Magalhaes MHCG (2001) Prevalence of numeric anomalies in the permanent dentition of patients with Down syndrome. Spec Care Dentist 21: 75-78.

62. Papadopoulos MA, Chatzoudi M, Kaklamanos EG (2010) Prevalence of Tooth Transposition. Angle Orthod 80: 275-285.

63. Shukla D, Bablani D, Chowdhry A, Thapar R, Gupta P, et al. (2014) Dentofacial and Cranial Changes in Down Syndrome. Osong Public Health and Research Perspectives 5: 339-344.

64. Somani R, Sunil MK, Jaidka S, Khaira J (2011) Dentofacial Anomalies and Oral Hygiene Status in Mentally Challenged Children: A Survey. J Indian Acad Oral Med Radiol 23: 51-53.

65. Russell BG, Kjaer I (1995) Tooth agenesis in Down syndrome. Am J Med Genet 55: 466-471.

66. Shapira J, Chaushu S, Becker A (2000) Prevalence of Tooth Transposition, Third Molar Agenesis, and Maxillary Canine Impaction in Individuals with Down Syndrome. Angle Orthod 70: 290-296.

67. Bauer D, Evans CA, BeGole EA, Salzmann L (2012) Severity of Occlusal Disharmonies in Down Syndrome. Int J Dent 2012: 872367.

68. Fink GB, Madaus WK, Walker GF (1975) A quantitative study of the face in Down's syndrome. Am J Orthod 67: 540-553.

69. Shapiro BL, Gorlin RJ, Redman RS, Bruhl HH (1967) The Palate and Down's Syndrome. N Engl J Med 276: 1460-1463.

70. Redman RS, Shapiro BL, Gorlin RJ (1965) Measurement of normal and reportedly malformed palatal vaults: III. Down's syndrome (trisomy 21, mongolism). J Pediatr 67: 162-165.

71. Oliveira AC, Pordeus IA, Torres CS, Martins MT, Paiva SM (2010) Feeding and nonnutritive sucking habits and prevalence of open bite and crossbite in children/adolescents with Down syndrome. Angle Orthod 80: 748-753.

72. Korayem MA, AlKofide EA (2014) Characteristics of Down sydrome subjects in a Saudi sample. Angle Orthod 84: 30-37.

73. Quintanilla J, Biedma B, Rodríguez M, Mora M, Cunqueiro M, et al. (2002) Cephalometrics in children with Down's syndrome. Pediatr Radiol 32: 635-643.

74. Townsend GC (1983) Fluctuating dental asymmetry in Down's syndrome. Australian Dental Journal 28: 39-44.

75. Macho V, Coelho A, Areias C, Macedo P, Andrade D (2014) Craniofacial features and specific oral characteristics of Down syndrome children. Oral Health and Dental Management 13: 408-411.

76. Witkop CJ, Keenan KM, Červenka J, Jaspers MT, Opitz JM, et al. (1988) Taurodontism: An anomaly of teeth reflecting disruptive developmental homeostasis. Am J Med Genet A 31: 85-97.

77. López-Pérez R, López-Morales P, Borges-Yáñez SA, Maupomé G, Parés-Vidrio G (2007) Prevalence of bruxism among Mexican children with Down syndrome. Downs Synd Res Pract 12: 45-49.

78. Bell EJ, Kaidonis J, Townsend GC (2002) Tooth Wear in Children with Down Syndrome. Aust Dent J 47: 30-35.

79. Smith BA, Ulrich BD (2008) Early onset of stabilizing strategies for gait and obstacles: Older adults with Down syndrome. Gait Posture 28: 448-455.

80. Pizzutillo PD, Herman MJ (2005) Cervical spine issues in Down syndrome. J Pediatr Orthop 25: 253-259.

81. Davidson RG (1988) Atlantoaxial instability in individuals with Down syndrome: a fresh look at the evidence. Pediatrics 81: 857-865.

82. Hata T, Todd MM (2005) Cervical spine considerations when anesthetizing patients with Down syndrome. Anesthesiology-Hagerstown 102: 680-686.

83. Browd S, Healy LJ, Dobie G, III JTJ, Jones GM, et al. (2006) Morphometric and qualitative analysis of congenital occipitocervical instability in children: implications for patients with Down syndrome. J Neurosurg Pediatr 105: 50-54.

84. Kaifu Y, Baba H, Sutikna T, Morwood MJ, Kubo D, et al. (2011) Craniofacial morphology of *Homo floresiensis*: Description, taxonomic affinities, and evolutionary implication. J Hum Evol 61: 644-682.

85. Concolino D, Pasquzzi A, Capalbo G, Sinopoli S, Strisciuglio P (2006) Early detection of podiatric anomalies in children with Down syndrome. Acta Paediatr 95: 17-20.

86. Lin C-J, Lai K-A, Kuan T-S, Chou Y-L (2001) Correlating factors and clinical significance of flexible flatfoot in preschool children. J Pediatr Orthop 21: 378-382.

87. Pfeiffer M, Kotz R, Ledl T, Hauser G, Sluga M (2006) Prevalence of flat foot in preschool-aged children. Pediatrics 118: 634-639.

88. Merrick J, Ezra. E, Josef B, Hendel D, Steinberg DM, et al. (2000) Musculoskeletal problems in Down syndrome. European Paediatric Orthopaedic Society survey: the Israeli sample. J Pediatr Orthop B 9: 185-192.

89. Ross CG, Shore S (2011) The effect of gross motor therapy and orthotic intervention in children with hypotonia and flexible flatfeet. JPO: Journal of Prosthetics and Orthotics 23: 149-154.

90. Lloyd-Roberts GC, Spence AJ (1958) Congenital vertical talus. J Bone Joint Surg Br 40-B: 33-41.

91. Furdon SA, Donlon CR (2002) Examination of the newborn foot: positional and structural abnormalities. Adv Neonatal Care 2: 248-258.

92. Jungers WL, Harcourt-Smith WEH, Wunderlich RE, Tocheri MW, Larson SG, et al. (2009) The foot of *Homo floresiensis*. Nature 459: 81-84.

93. Jungers WL, Larson SG, Harcourt-Smith W, Morwood MJ, Sutikna T, et al. (2009) Descriptions of the lower limb skeleton of *Homo floresiensis*. J Hum Evol 57: 538-554.

94. Harcourt-Smith WEH, Aiello LC (2004) Fossils, feet and the evolution of human bipedal locomotion. J Anat 204: 403-416.

95. Coughlin MJ, Jones CP (2007) Hallux valgus: demographics, etiology, and radiographic assessment. Foot & Ankle International 28: 759-777.

96. Kilmartin TE, Barrington RL, Wallace WA (1991) Metatarsus primus varus: a statistical study. J Bone Joint Surg [Br] 73: 937-940.

97. Arden N, Nevitt MC (2006) Osteoarthritis: Epidemiology. Best Practice & Research Clinical Rheumatology 20: 3-25.
